# Supplementary material for: Meta-analysis identifies five novel loci associated with endometriosis highlighting key genes involved in hormone metabolism
Source: Nat Commun. 2017 May 24;8:15539. doi: 10.1038/ncomms15539 (PMC5458088; doi:10.1038/ncomms15539)
Supplement: Supplementary Information — Supplementary Figures, Supplementary Notes and Supplementary References [file ncomms15539-s1.pdf]

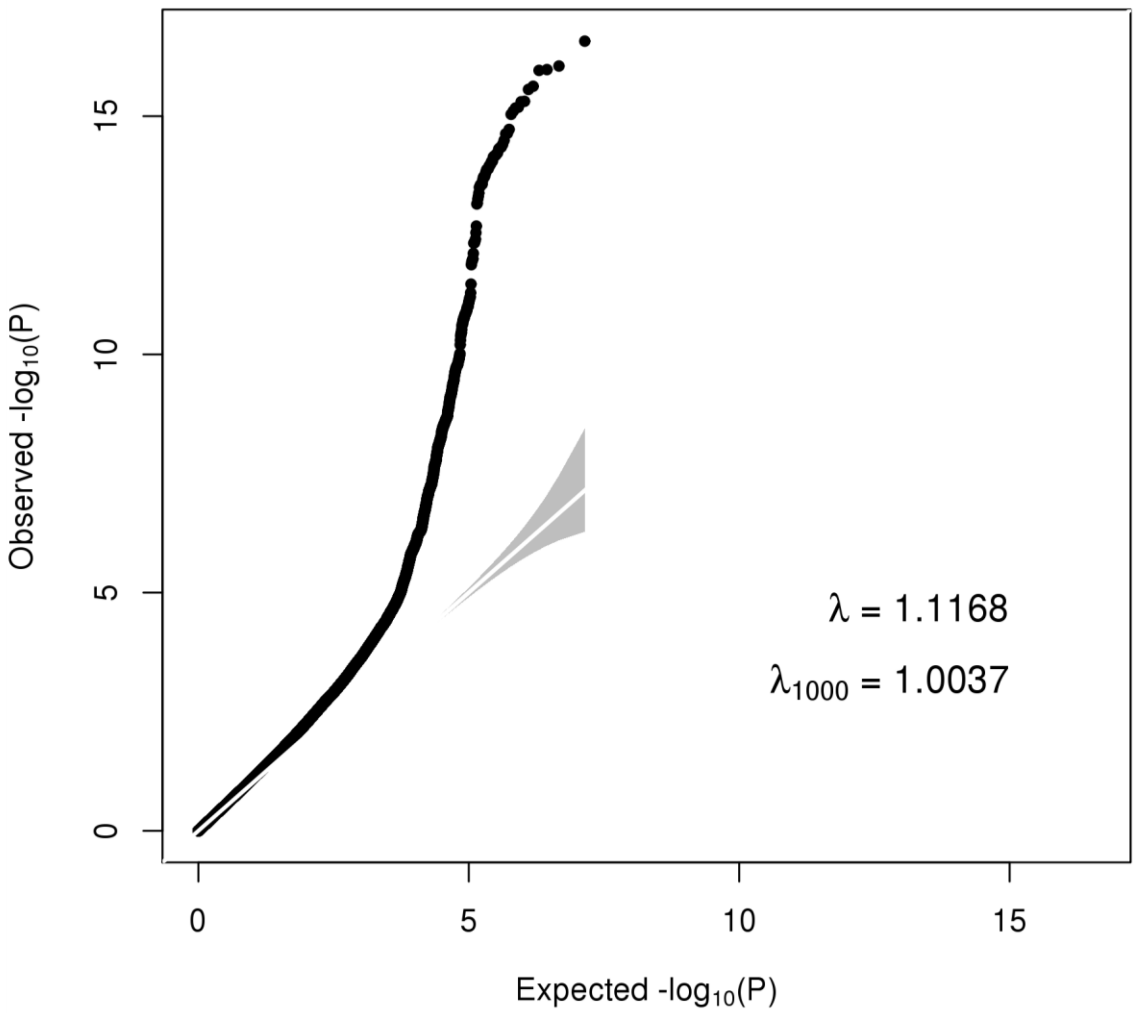

Supplementary Figure 1: Q-Q plot for the GWA meta-analysis of all endometriosis cases versus controls in the QIMRHCS, deCODE, LEUVEN, OX, 23andMe, NHS2-dbGaP, WGHS, BBJ, Adachi-500K, Adachi-6 and iPSYCH datasets including all autosomal SNPs (black data points). The plot was constructed by ranking *P* values from smallest to largest (the 'order' statistics) and plotting them against their expected values under the null hypothesis of no association (samples from the known chi-squared distribution). Deviations above the line of equality (drawn in white) indicate a preponderance of smaller *P* values. To aid interpretation, we have also calculated 95% confidence envelopes (shaded grey). These are formed by calculating, for reach order statistic, the 2.5th and 97.5th centiles of the distribution of the order statistic under random sampling and the null hypothesis. The genomic inflation factors (λ) are also shown, defined as the ratio of the median of the empirically observed distribution of the test statistic to the expected median, thus quantifying the extent of the bulk inflation. The raw λ, and the λ for an equivalent study of 1000 cases and 1000 controls (λ_{1000}) indicate these data have no undetected sample duplications, unknown familial relationships, systematic technical bias or gross (uncorrected) population stratification.

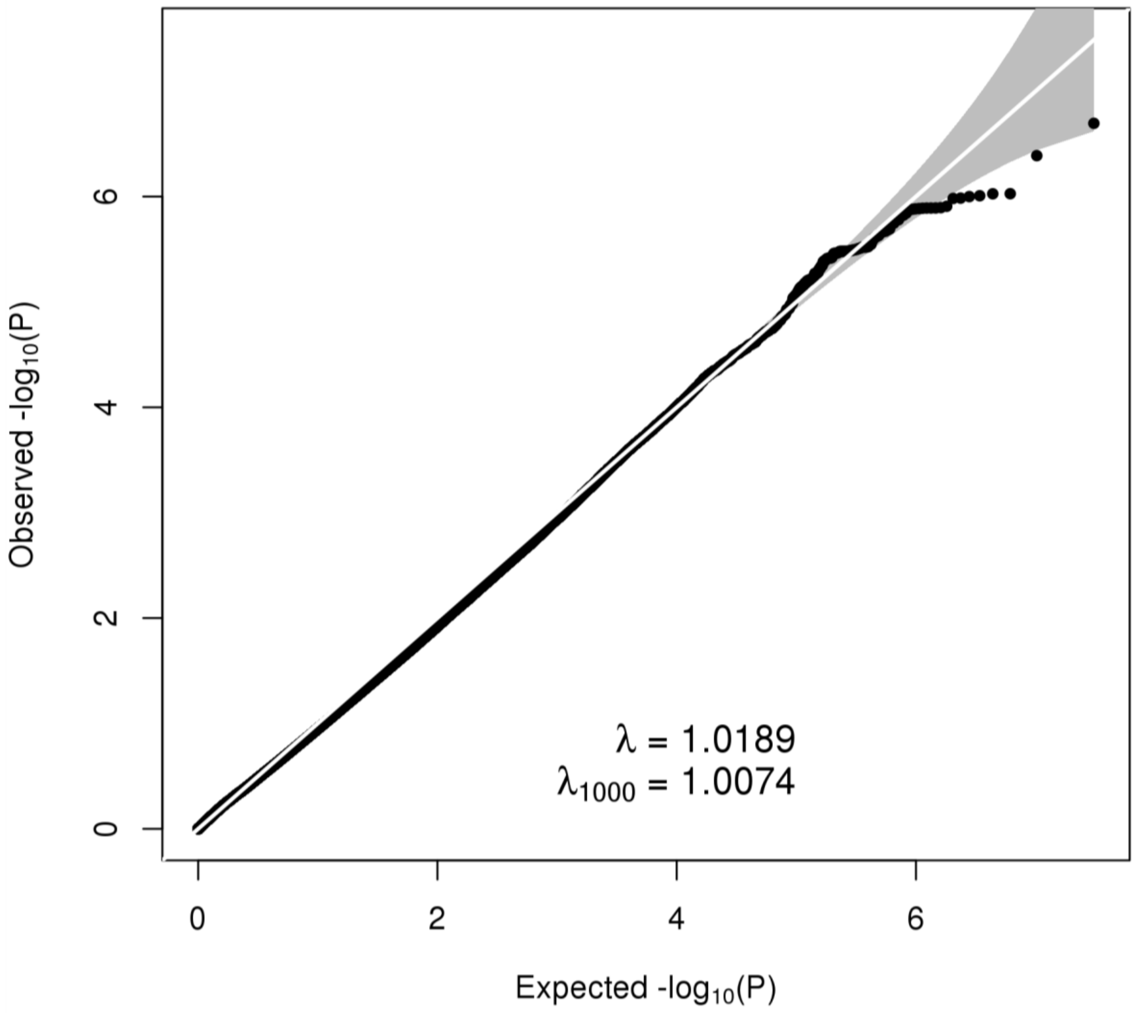

Supplementary Figure 2: Q-Q plot for the QIMRHCS 1000 Genomes-imputed GWA datasets including all autosomal SNPs (black data points). The plot was constructed by ranking *P* values from smallest to largest (the 'order' statistics) and plotting them against their expected values under the null hypothesis of no association (samples from the known chi-squared distribution). Deviations above the line of equality (drawn in white) indicate a preponderance of smaller *P* values. To aid interpretation, we have also calculated 95% confidence envelopes (shaded grey). These are formed by calculating, for each order statistic, the 2.5th and 97.5th centiles of the distribution of the order statistic under random sampling and the null hypothesis. The genomic inflation factors (λ) are also shown, defined as the ratio of the median of the empirically observed distribution of the test statistic to the expected median, thus quantifying the extent of the bulk inflation. The raw λ, and the λ for an equivalent study of 1000 cases and 1000 controls (λ_{1000}) indicate these data have no undetected sample duplications, unknown familial relationships, systematic technical bias or gross (uncorrected) population stratification.

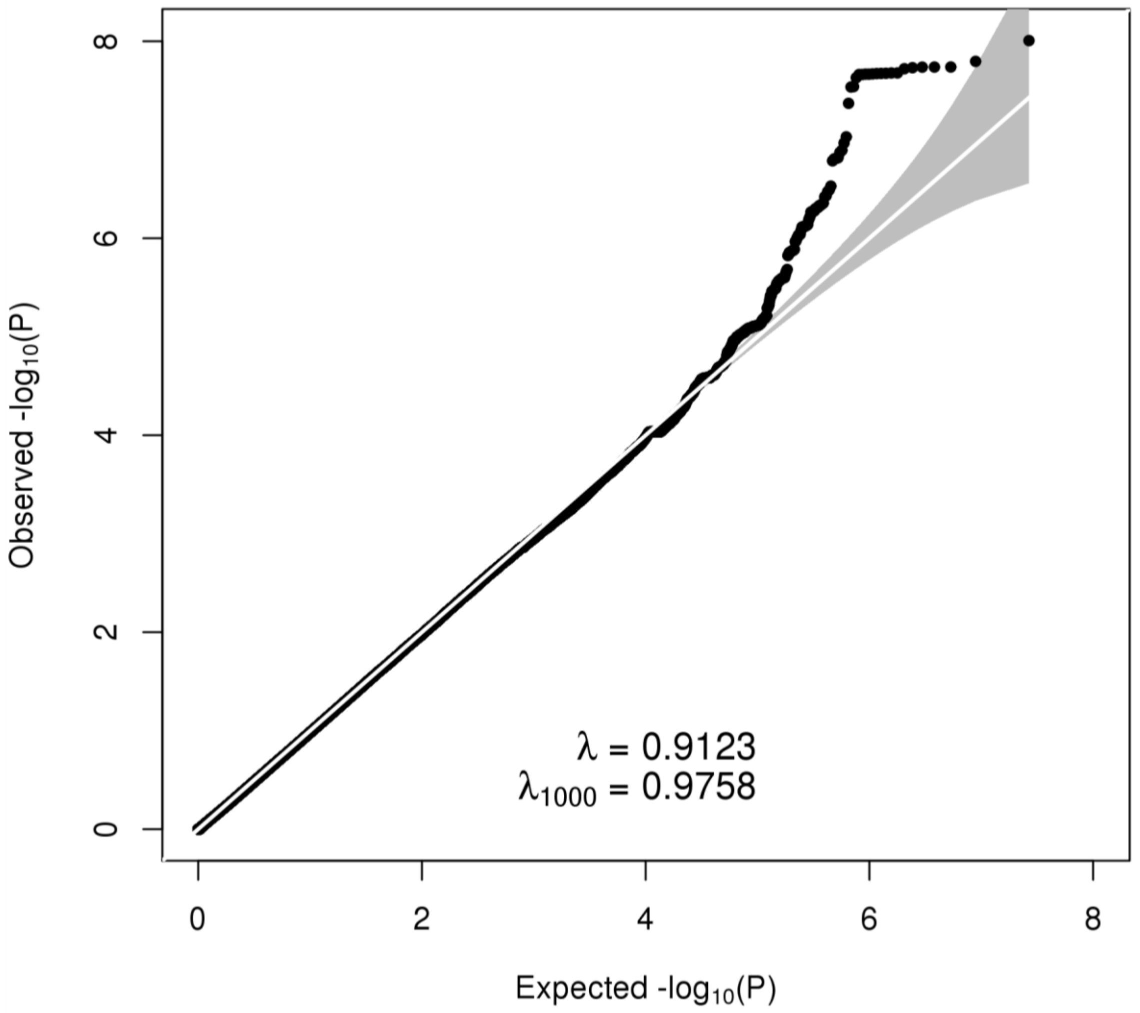

Supplementary Figure 3: Q-Q plot for the deCODE 1000 Genomes-imputed GWA datasets including all autosomal SNPs (black data points). The plot was constructed by ranking *P* values from smallest to largest (the 'order' statistics) and plotting them against their expected values under the null hypothesis of no association (samples from the known chi-squared distribution). Deviations above the line of equality (drawn in white) indicate a preponderance of smaller *P* values. To aid interpretation, we have also calculated 95% confidence envelopes (shaded grey). These are formed by calculating, for each order statistic, the 2.5th and 97.5th centiles of the distribution of the order statistic under random sampling and the null hypothesis. The genomic inflation factors (λ) are also shown, defined as the ratio of the median of the empirically observed distribution of the test statistic to the expected median, thus quantifying the extent of the bulk inflation. The raw λ, and the λ for an equivalent study of 1000 cases and 1000 controls (λ_{1000}) indicate these data have no undetected sample duplications, unknown familial relationships, systematic technical bias or gross (uncorrected) population stratification.

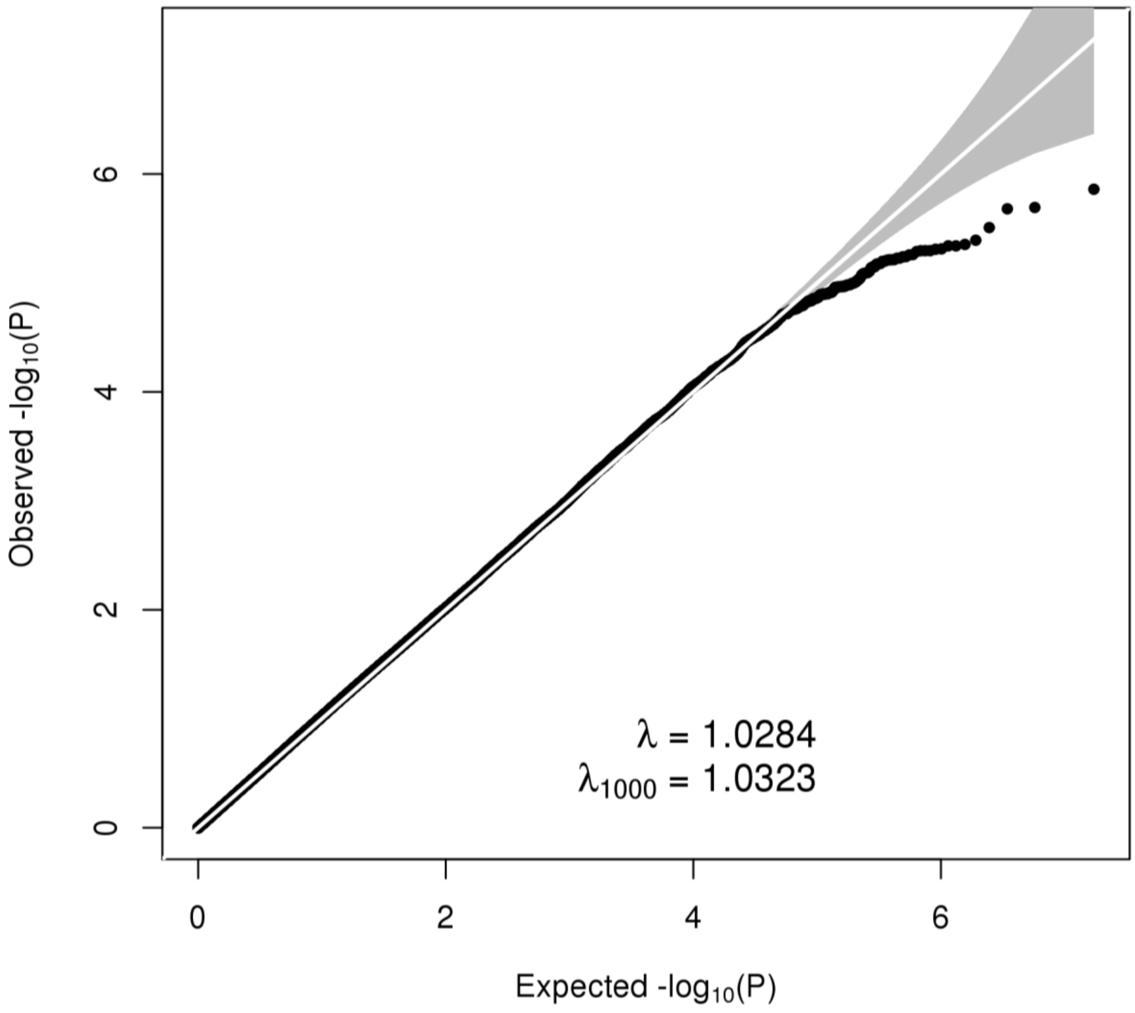

Supplementary Figure 4: Q-Q plot for the LEUVEN 1000 Genomes-imputed GWA datasets including all autosomal SNPs (black data points). The plot was constructed by ranking *P* values from smallest to largest (the 'order' statistics) and plotting them against their expected values under the null hypothesis of no association (samples from the known chi-squared distribution). Deviations above the line of equality (drawn in white) indicate a preponderance of smaller *P* values. To aid interpretation, we have also calculated 95% confidence envelopes (shaded grey). These are formed by calculating, for each order statistic, the 2.5^{th} and 97.5^{th} centiles of the distribution of the order statistic under random sampling and the null hypothesis. The genomic inflation factors (λ) are also shown, defined as the ratio of the median of the empirically observed distribution of the test statistic to the expected median, thus quantifying the extent of the bulk inflation. The raw λ, and the λ for an equivalent study of 1000 cases and 1000 controls (λ_{1000}) indicate these data have no undetected sample duplications, unknown familial relationships, systematic technical bias or gross (uncorrected) population stratification.

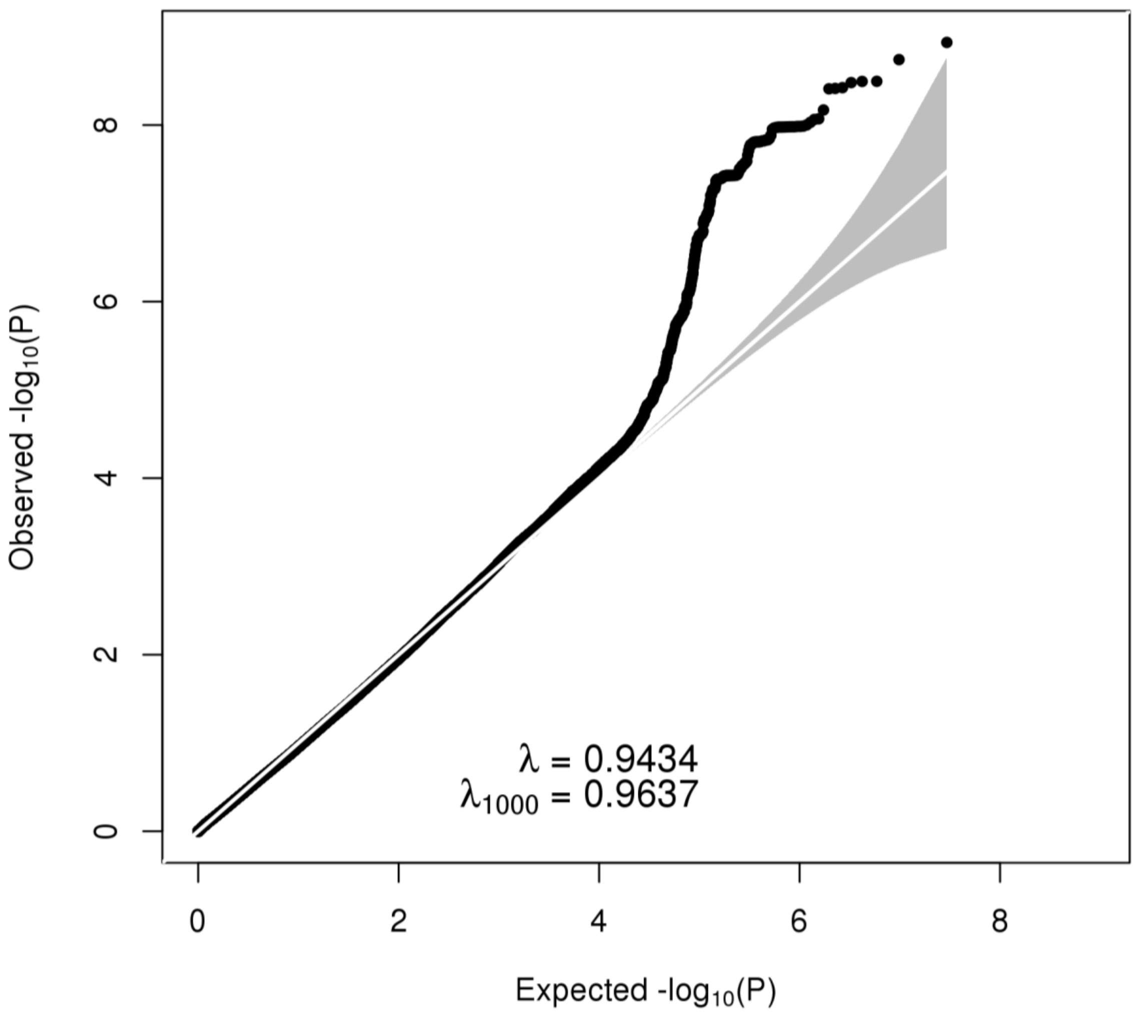

Supplementary Figure 5: Q-Q plot for the OX 1000 Genomes-imputed GWA datasets including all autosomal SNPs (black data points). The plot was constructed by ranking *P* values from smallest to largest (the 'order' statistics) and plotting them against their expected values under the null hypothesis of no association (samples from the known chi-squared distribution). Deviations above the line of equality (drawn in white) indicate a preponderance of smaller *P* values. To aid interpretation, we have also calculated 95% confidence envelopes (shaded grey). These are formed by calculating, for each order statistic, the 2.5th and 97.5th centiles of the distribution of the order statistic under random sampling and the null hypothesis. The genomic inflation factors (λ) are also shown, defined as the ratio of the median of the empirically observed distribution of the test statistic to the expected median, thus quantifying the extent of the bulk inflation. The raw λ, and the λ for an equivalent study of 1000 cases and 1000 controls (λ_{1000}) indicate these data have no undetected sample duplications, unknown familial relationships, systematic technical bias or gross (uncorrected) population stratification.

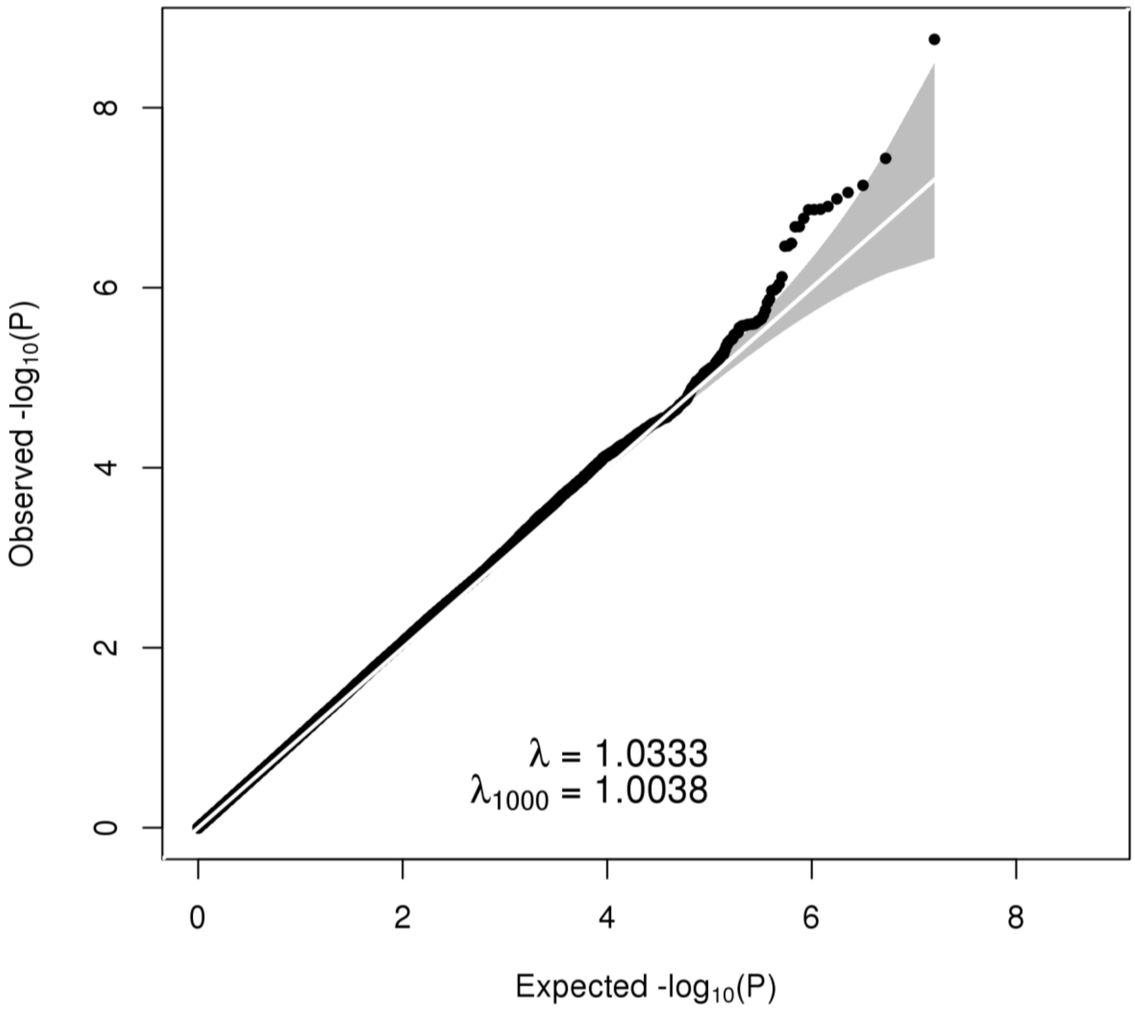

Supplementary Figure 6: Q-Q plot for the 23andMe 1000 Genomes-imputed GWA datasets including all autosomal SNPs (black data points). The plot was constructed by ranking *P* values from smallest to largest (the 'order' statistics) and plotting them against their expected values under the null hypothesis of no association (samples from the known chi-squared distribution). Deviations above the line of equality (drawn in white) indicate a preponderance of smaller *P* values. To aid interpretation, we have also calculated 95% confidence envelopes (shaded grey). These are formed by calculating, for each order statistic, the 2.5th and 97.5th centiles of the distribution of the order statistic under random sampling and the null hypothesis. The genomic inflation factors (λ) are also shown, defined as the ratio of the median of the empirically observed distribution of the test statistic to the expected median, thus quantifying the extent of the bulk inflation. The raw λ, and the λ for an equivalent study of 1000 cases and 1000 controls (λ_{1000}) indicate these data have no undetected sample duplications, unknown familial relationships, systematic technical bias or gross (uncorrected) population stratification.

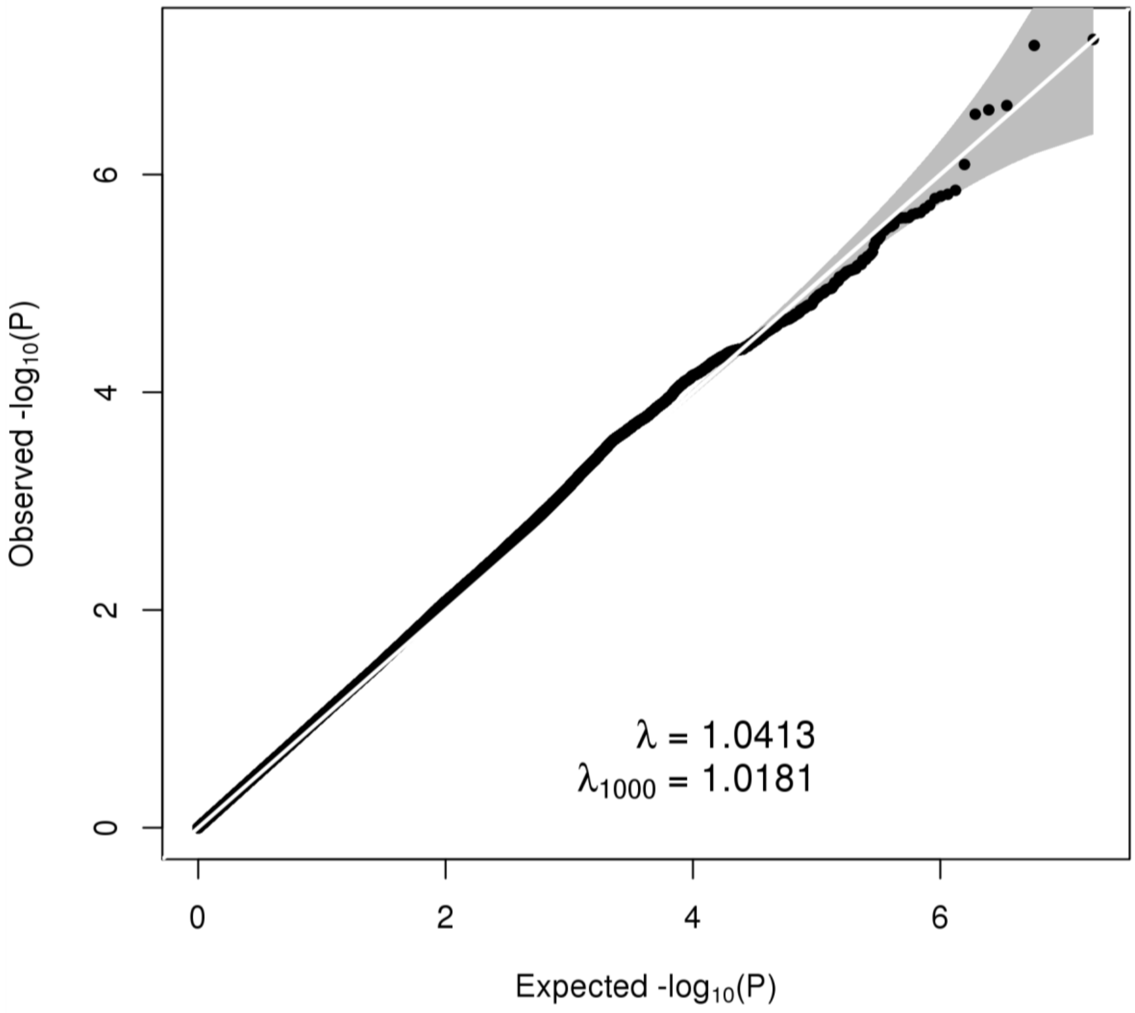

Supplementary Figure 7: Q-Q plot for the NHS2-dbGaP 1000 Genomes-imputed GWA datasets including all autosomal SNPs (black data points). The plot was constructed by ranking *P* values from smallest to largest (the 'order' statistics) and plotting them against their expected values under the null hypothesis of no association (samples from the known chi-squared distribution). Deviations above the line of equality (drawn in white) indicate a preponderance of smaller *P* values. To aid interpretation, we have also calculated 95% confidence envelopes (shaded grey). These are formed by calculating, for each order statistic, the 2.5th and 97.5th centiles of the distribution of the order statistic under random sampling and the null hypothesis. The genomic inflation factors (λ) are also shown, defined as the ratio of the median of the empirically observed distribution of the test statistic to the expected median, thus quantifying the extent of the bulk inflation. The raw λ, and the λ for an equivalent study of 1000 cases and 1000 controls (λ_{1000}) indicate these data have no undetected sample duplications, unknown familial relationships, systematic technical bias or gross (uncorrected) population stratification.

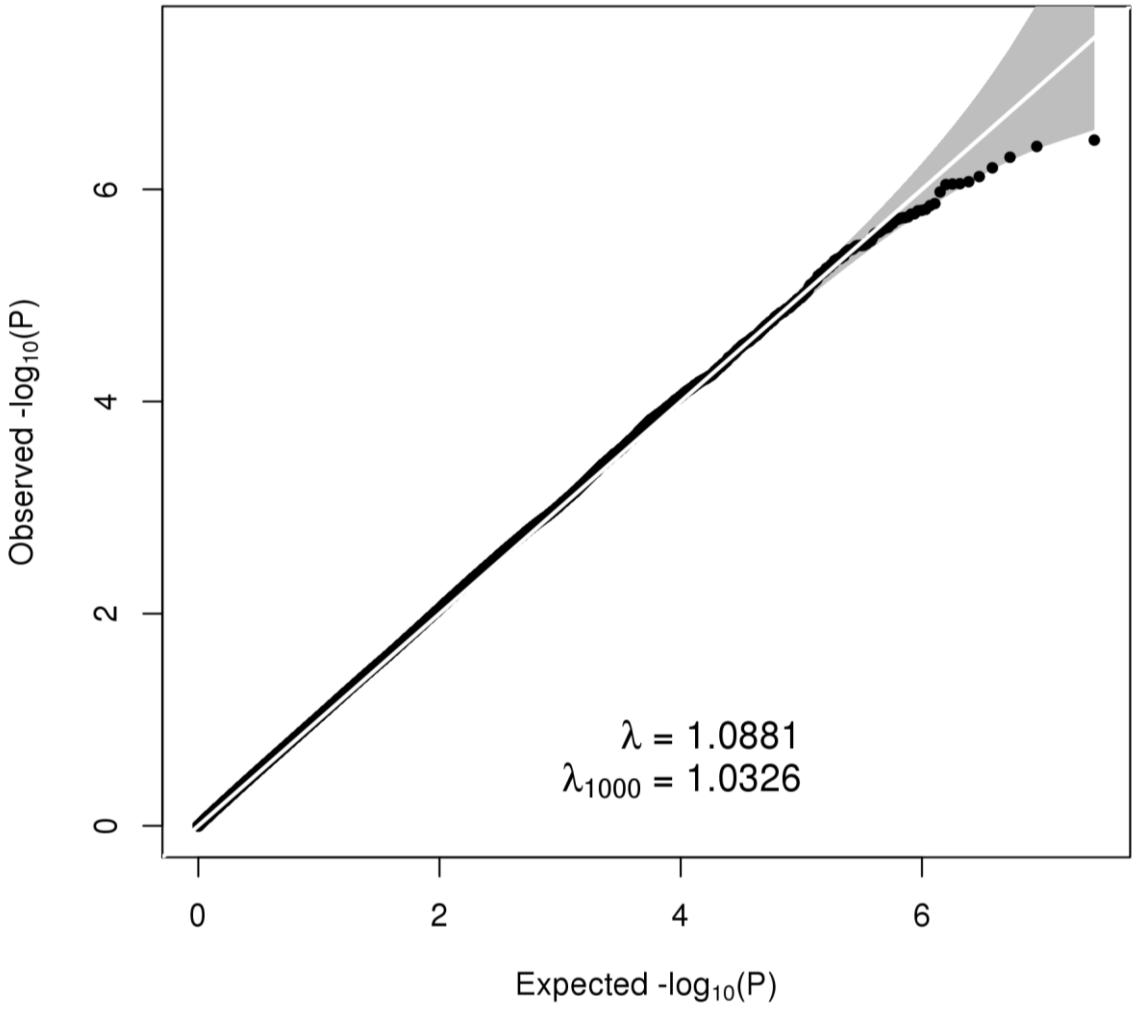

Supplementary Figure 8: Q-Q plot for the WGHS 1000 Genomes-imputed GWA datasets including all autosomal SNPs (black data points). The plot was constructed by ranking *P* values from smallest to largest (the 'order' statistics) and plotting them against their expected values under the null hypothesis of no association (samples from the known chi-squared distribution). Deviations above the line of equality (drawn in white) indicate a preponderance of smaller *P* values. To aid interpretation, we have also calculated 95% confidence envelopes (shaded grey). These are formed by calculating, for each order statistic, the 2.5^{th} and 97.5^{th} centiles of the distribution of the order statistic under random sampling and the null hypothesis. The genomic inflation factors (λ) are also shown, defined as the ratio of the median of the empirically observed distribution of the test statistic to the expected median, thus quantifying the extent of the bulk inflation. The raw λ, and the λ for an equivalent study of 1000 cases and 1000 controls (λ_{1000}) indicate these data have no undetected sample duplications, unknown familial relationships, systematic technical bias or gross (uncorrected) population stratification.

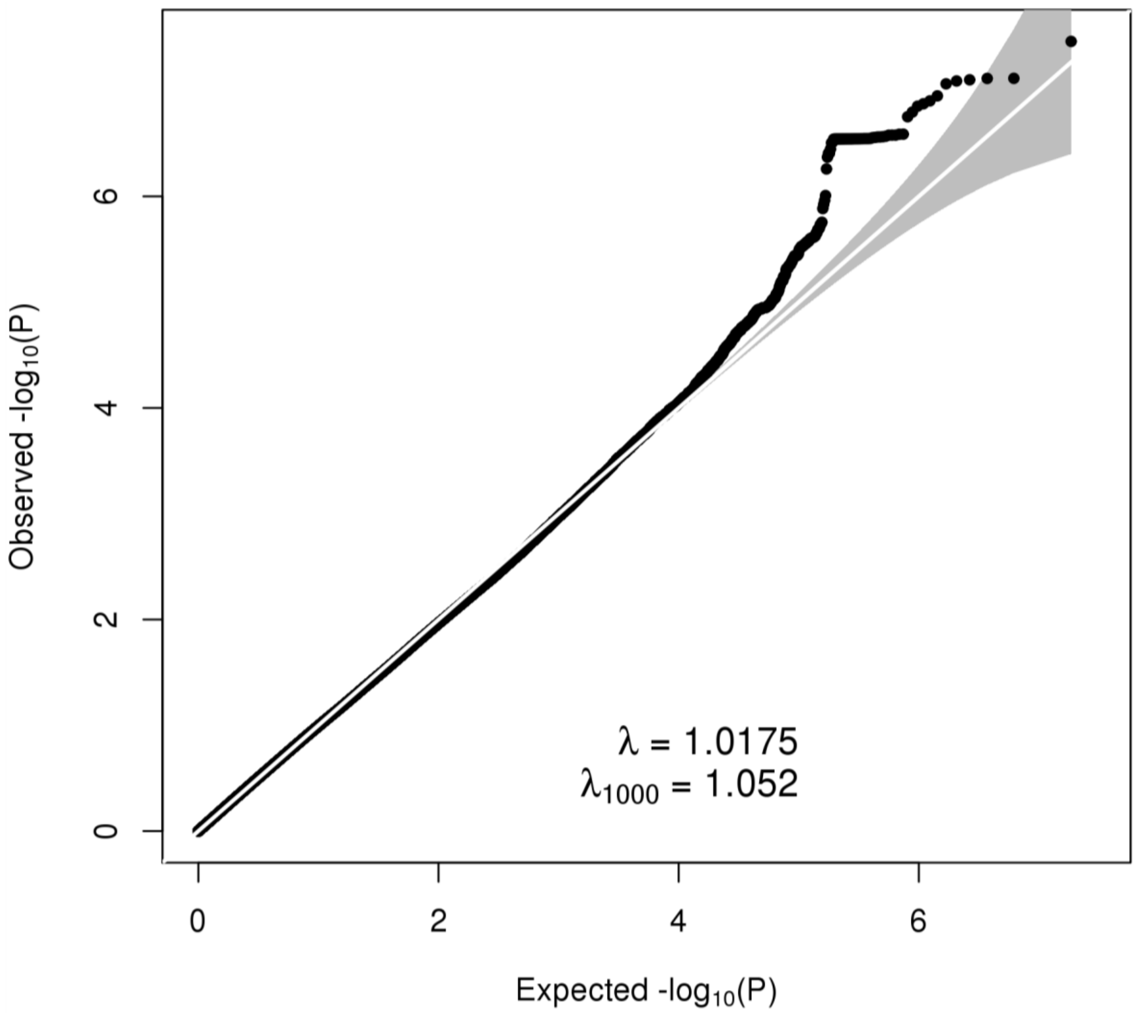

Supplementary Figure 9: Q-Q plot for the iPSYCH 1000 Genomes-imputed GWA datasets including all autosomal SNPs (black data points). The plot was constructed by ranking *P* values from smallest to largest (the 'order' statistics) and plotting them against their expected values under the null hypothesis of no association (samples from the known chi-squared distribution). Deviations above the line of equality (drawn in white) indicate a preponderance of smaller *P* values. To aid interpretation, we have also calculated 95% confidence envelopes (shaded grey). These are formed by calculating, for reach order statistic, the 2.5th and 97.5th centiles of the distribution of the order statistic under random sampling and the null hypothesis. The genomic inflation factors (λ) are also shown, defined as the ratio of the median of the empirically observed distribution of the test statistic to the expected median, thus quantifying the extent of the bulk inflation. The raw λ, and the λ for an equivalent study of 1000 cases and 1000 controls (λ_{1000}) indicate these data have no undetected sample duplications, unknown familial relationships, systematic technical bias or gross (uncorrected) population stratification.

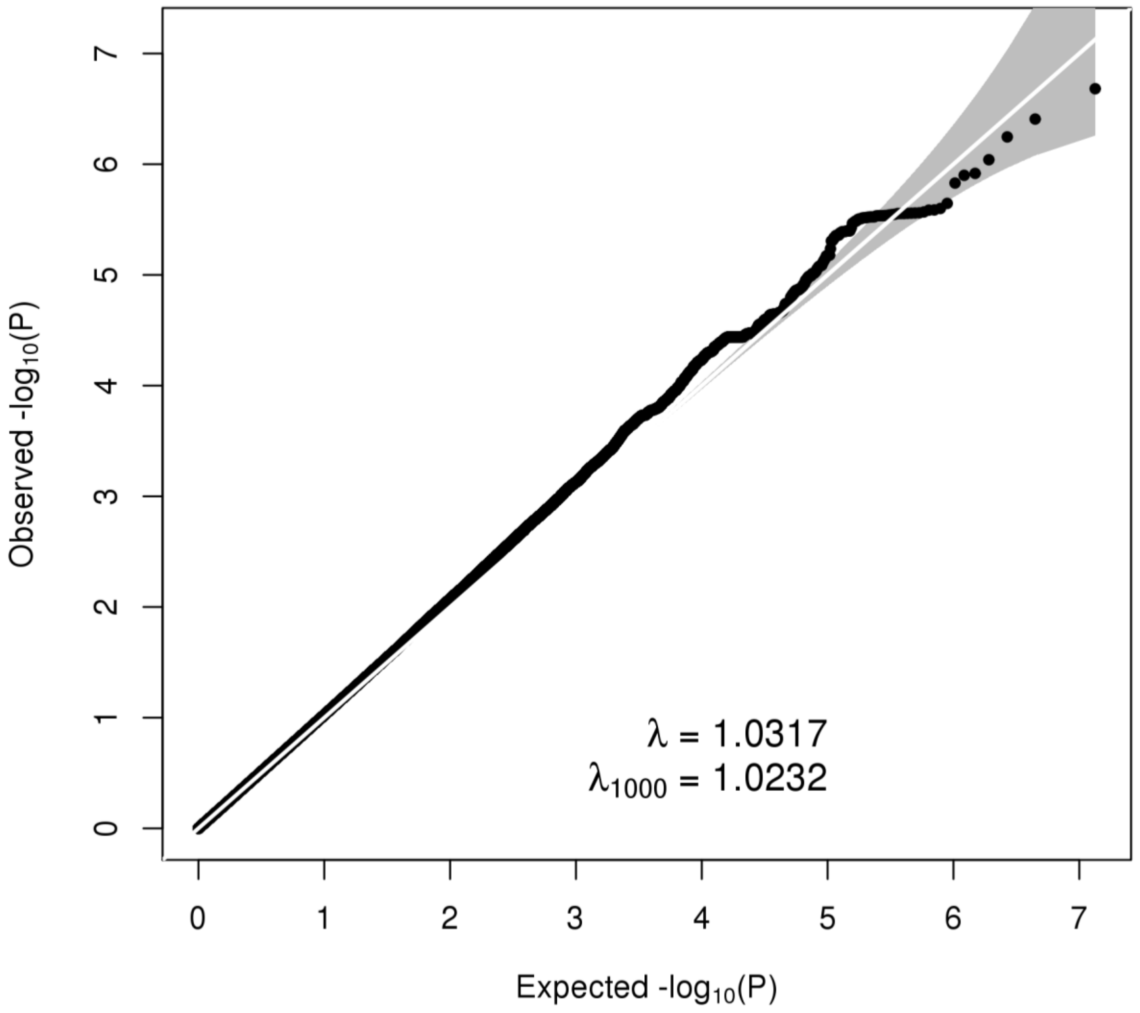

Supplementary Figure 10: Q-Q plot for the BBJ 1000 Genomes-imputed GWA datasets including all autosomal SNPs (black data points). The plot was constructed by ranking *P* values from smallest to largest (the 'order' statistics) and plotting them against their expected values under the null hypothesis of no association (samples from the known chi-squared distribution). Deviations above the line of equality (drawn in white) indicate a preponderance of smaller *P* values. To aid interpretation, we have also calculated 95% confidence envelopes (shaded grey). These are formed by calculating, for each order statistic, the 2.5th and 97.5th centiles of the distribution of the order statistic under random sampling and the null hypothesis. The genomic inflation factors (λ) are also shown, defined as the ratio of the median of the empirically observed distribution of the test statistic to the expected median, thus quantifying the extent of the bulk inflation. The raw λ, and the λ for an equivalent study of 1000 cases and 1000 controls (λ_{1000}) indicate these data have no undetected sample duplications, unknown familial relationships, systematic technical bias or gross (uncorrected) population stratification.

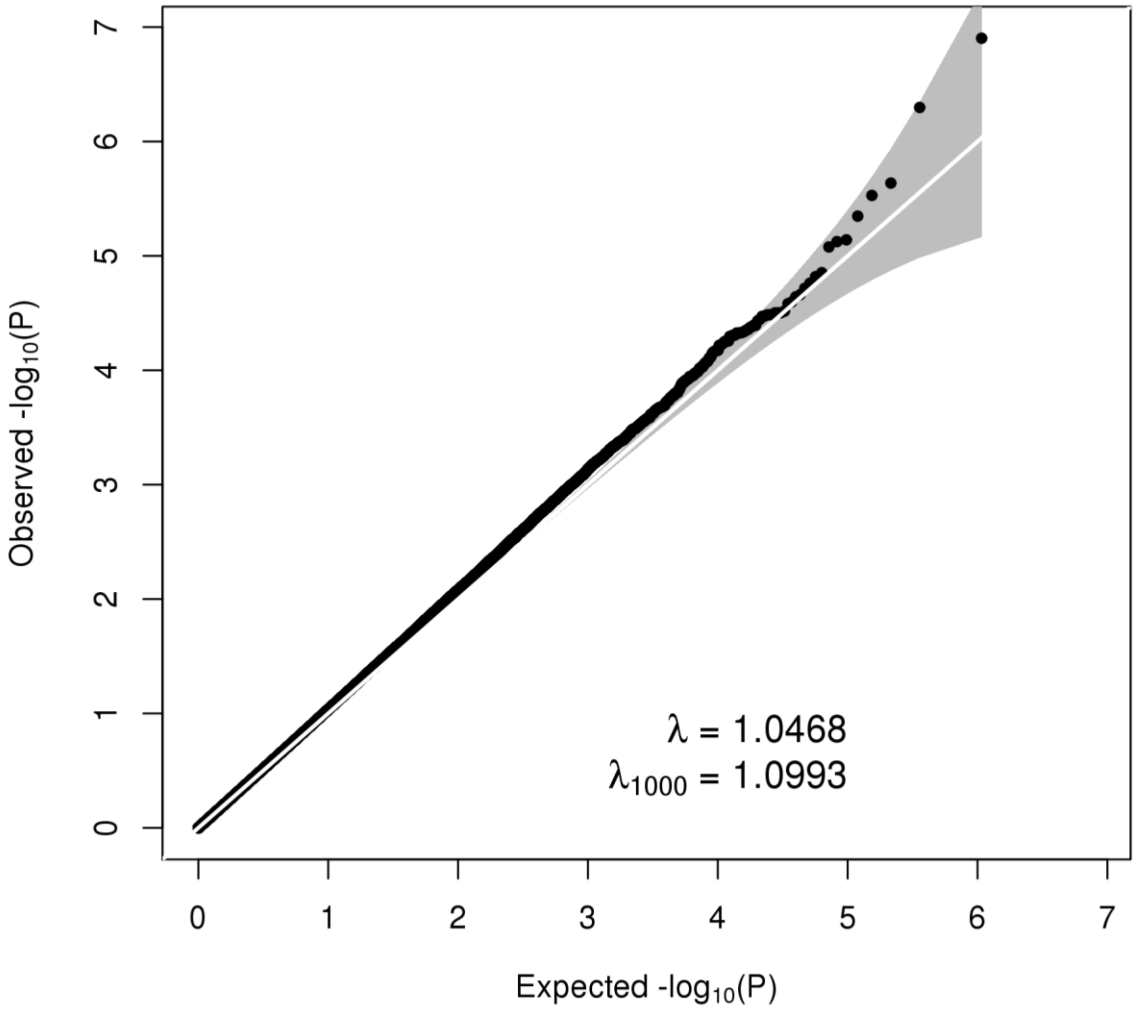

Supplementary Figure 11: Q-Q plot for the Adachi-6 observed GWA datasets including all autosomal SNPs (black data points). The plot was constructed by ranking *P* values from smallest to largest (the 'order' statistics) and plotting them against their expected values under the null hypothesis of no association (samples from the known chi-squared distribution). Deviations above the line of equality (drawn in white) indicate a preponderance of smaller *P* values. To aid interpretation, we have also calculated 95% confidence envelopes (shaded grey). These are formed by calculating, for each order statistic, the 2.5th and 97.5th centiles of the distribution of the order statistic under random sampling and the null hypothesis. The genomic inflation factors (λ) are also shown, defined as the ratio of the median of the empirically observed distribution of the test statistic to the expected median, thus quantifying the extent of the bulk inflation. The raw λ, and the λ for an equivalent study of 1000 cases and 1000 controls (λ_{1000}) indicate these data have no undetected sample duplications, unknown familial relationships, systematic technical bias or gross (uncorrected) population stratification.

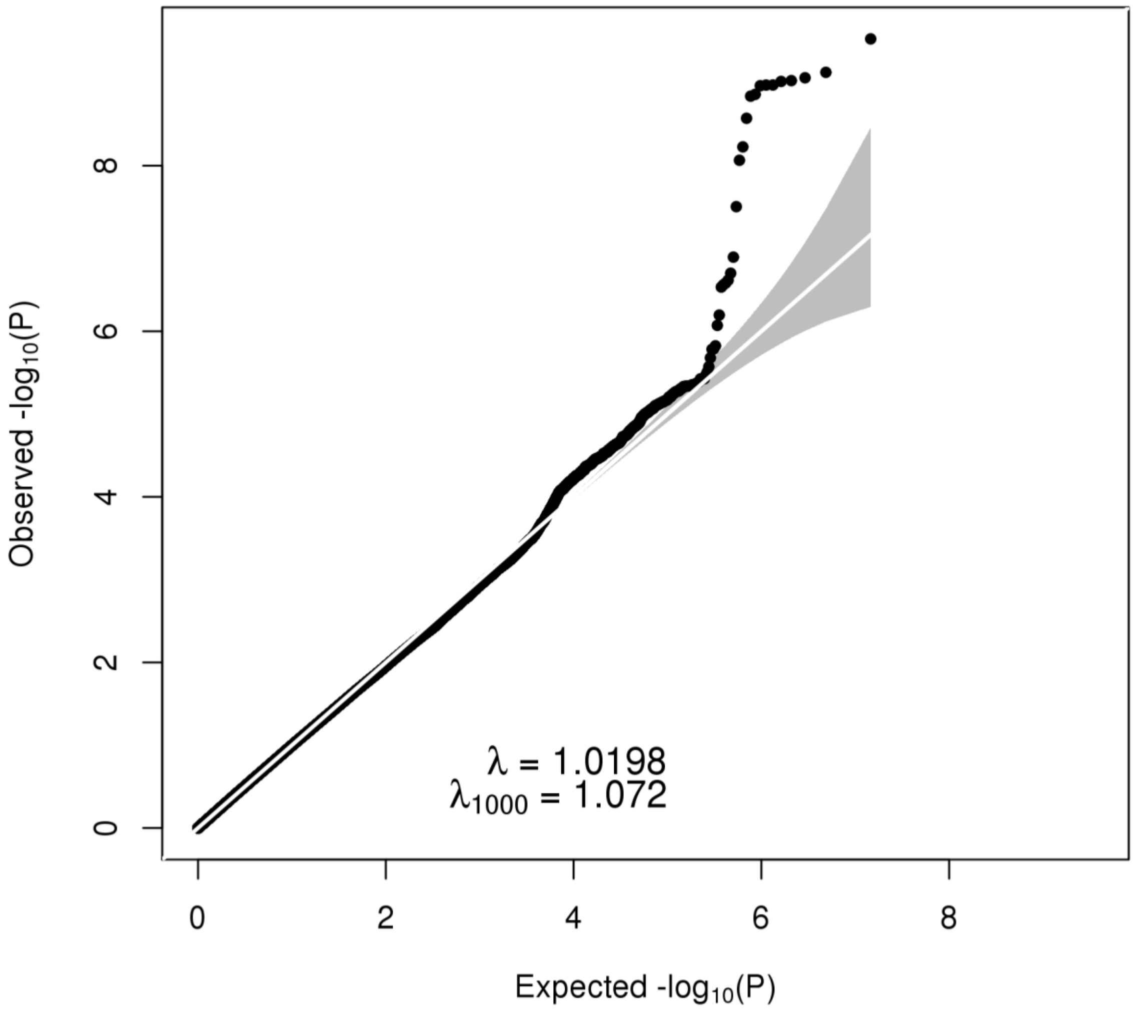

Supplementary Figure 12: Q-Q plot for the Adachi-500K 1000 Genomes-imputed GWA datasets including all autosomal SNPs (black data points). The plot was constructed by ranking *P* values from smallest to largest (the 'order' statistics) and plotting them against their expected values under the null hypothesis of no association (samples from the known chi-squared distribution). Deviations above the line of equality (drawn in white) indicate a preponderance of smaller *P* values. To aid interpretation, we have also calculated 95% confidence envelopes (shaded grey). These are formed by calculating, for each order statistic, the 2.5^{th} and 97.5^{th} centiles of the distribution of the order statistic under random sampling and the null hypothesis. The genomic inflation factors (λ) are also shown, defined as the ratio of the median of the empirically observed distribution of the test statistic to the expected median, thus quantifying the extent of the bulk inflation. The raw λ, and the λ for an equivalent study of 1000 cases and 1000 controls (λ_{1000}) indicate these data have no undetected sample duplications, unknown familial relationships, systematic technical bias or gross (uncorrected) population stratification.

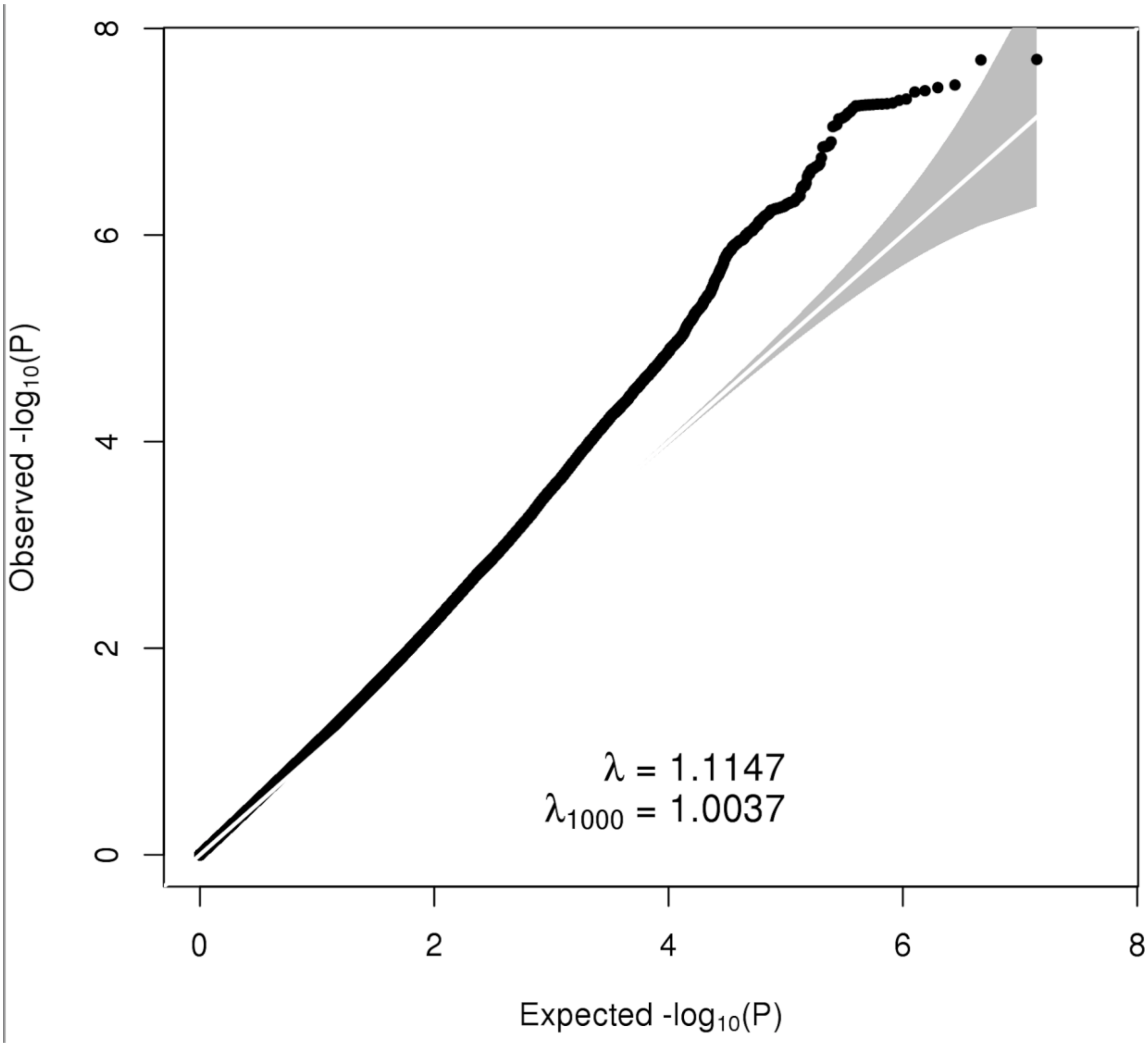

Supplementary Figure 13: Q-Q plot for the fixed-effects GWA meta-analysis of all endometriosis cases versus controls in the combined QIMRHCS+OX, deCODE, LEUVEN, 23andMe, NHS2-dbGaP, WGHS, BBJ, Adachi-500K, Adachi-6 and iPSYCH datasets including all autosomal SNPs (black data points) but excluding all SNPs within the nine previously identified risk loci. The plot was constructed by ranking *P* values from smallest to largest (the 'order' statistics) and plotting them against their expected values under the null hypothesis of no association (samples from the known chi-squared distribution). Deviations above the line of equality (drawn in white) indicate a preponderance of smaller *P* values. To aid interpretation, we have also calculated 95% confidence envelopes (shaded grey). These are formed by calculating, for reach order statistic, the 2.5^{th} and 97.5^{th} centiles of the distribution of the order statistic under random sampling and the null hypothesis. The genomic inflation factors (λ) are also shown, defined as the ratio of the median of the empirically observed distribution of the test statistic to the expected median, thus quantifying the extent of the bulk inflation. The raw λ, and the λ for an equivalent study of 1000 cases and 1000 controls (λ_{1000}) indicate these data have no undetected sample duplications, unknown familial relationships, systematic technical bias or gross (uncorrected) population stratification.

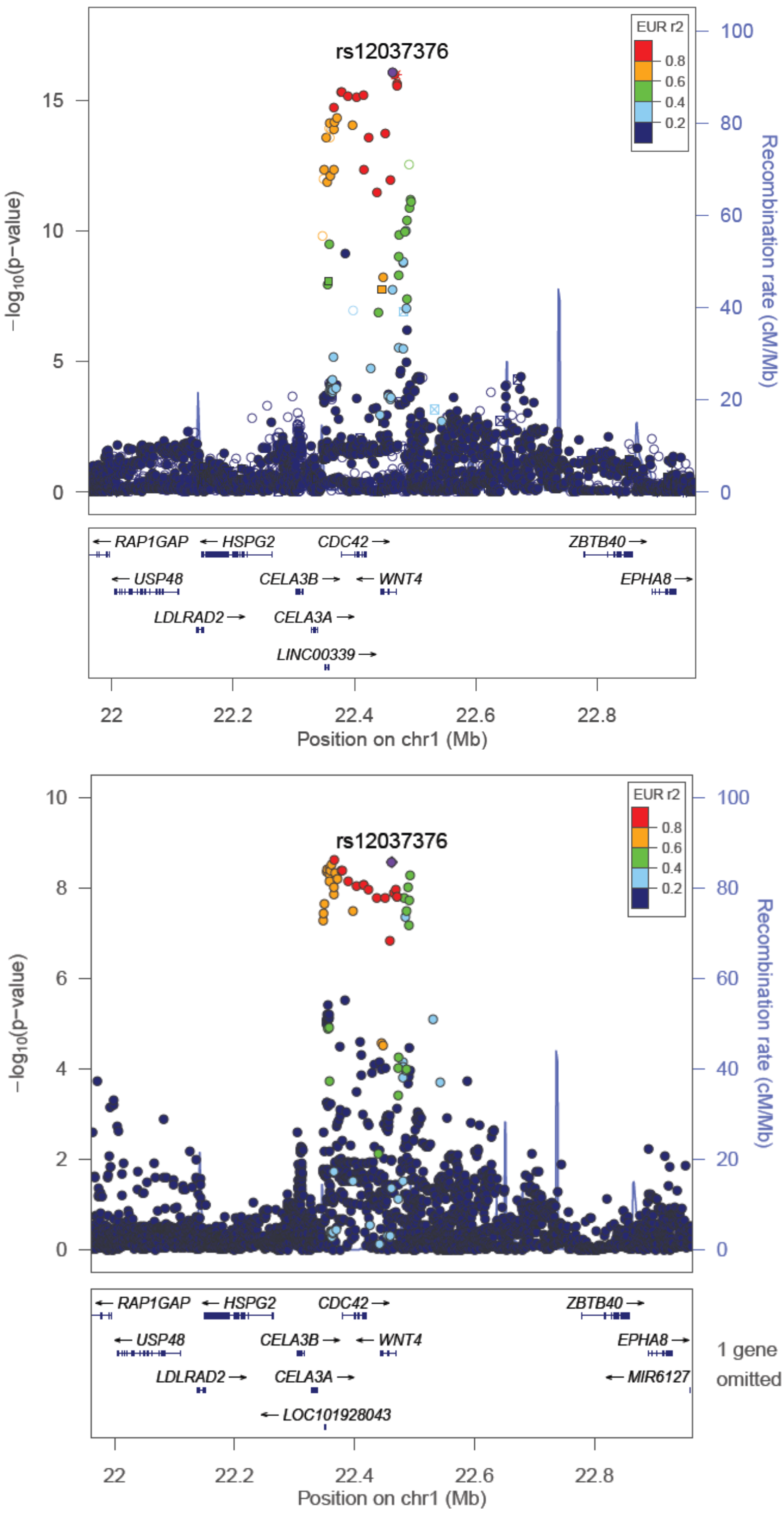

Supplementary Figure 14 | Evidence of association from the fixed-effects GWA meta-analysis including all (top figure) and Grade B (bottom figure) endometriosis cases across the 1p36.12 region. SNPs are shown as circles, diamonds or squares (filled or unfilled), with the top SNP represented by purple color. All other SNPs are color coded according to the strength of LD with the top SNP (as measured by r^2 in the European 1000 Genomes data).

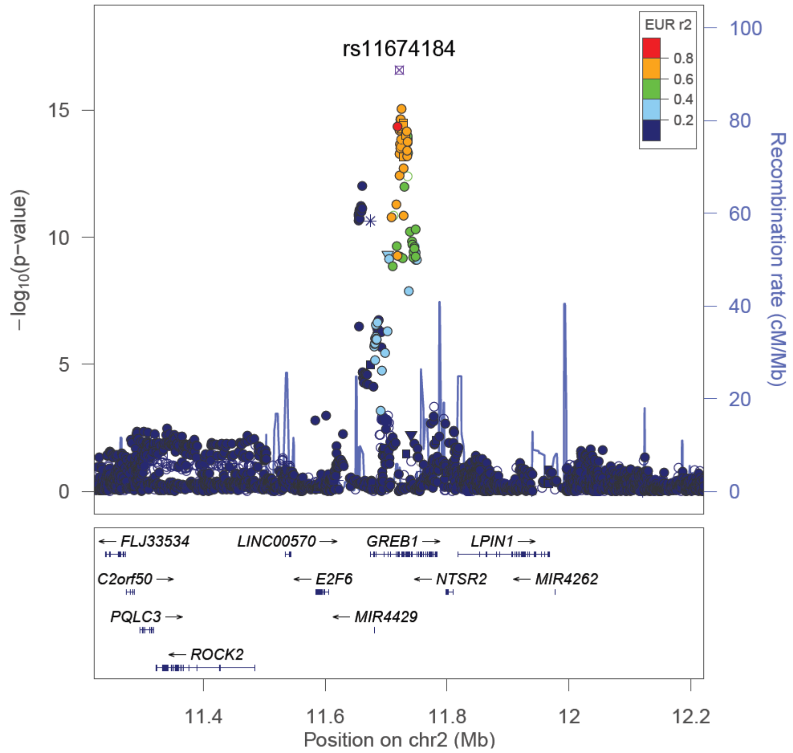

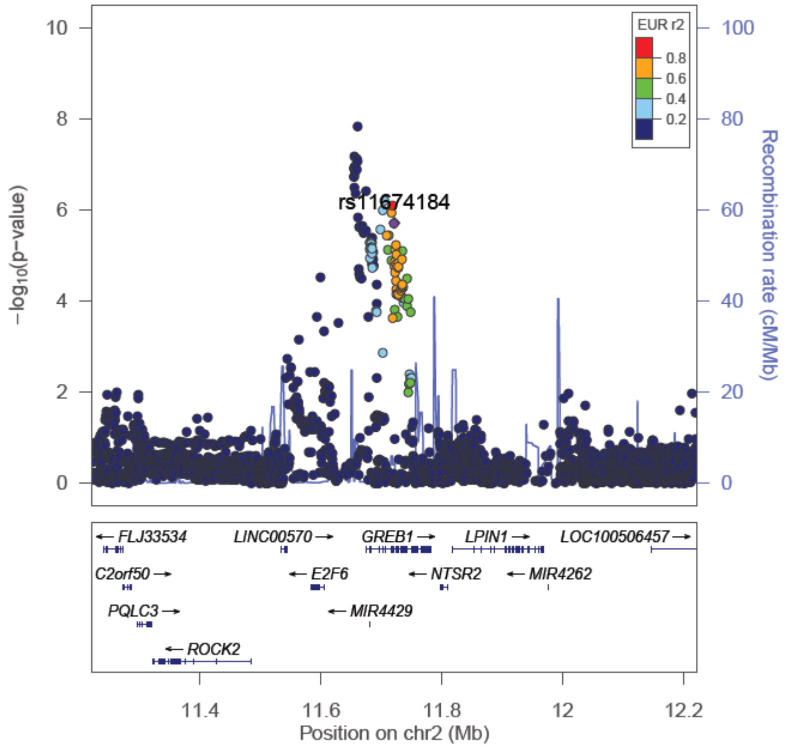

Supplementary Figure 15 | Evidence of association from the fixed-effects GWA meta-analysis including all (top figure) and Grade B (bottom figure) endometriosis cases across the 2p25.1 region. SNPs are shown as circles, diamonds or squares (filled or unfilled), with the top SNP represented by purple color. All other SNPs are color coded according to the strength of LD with the top SNP (as measured by r^2 in the European 1000 Genomes data).

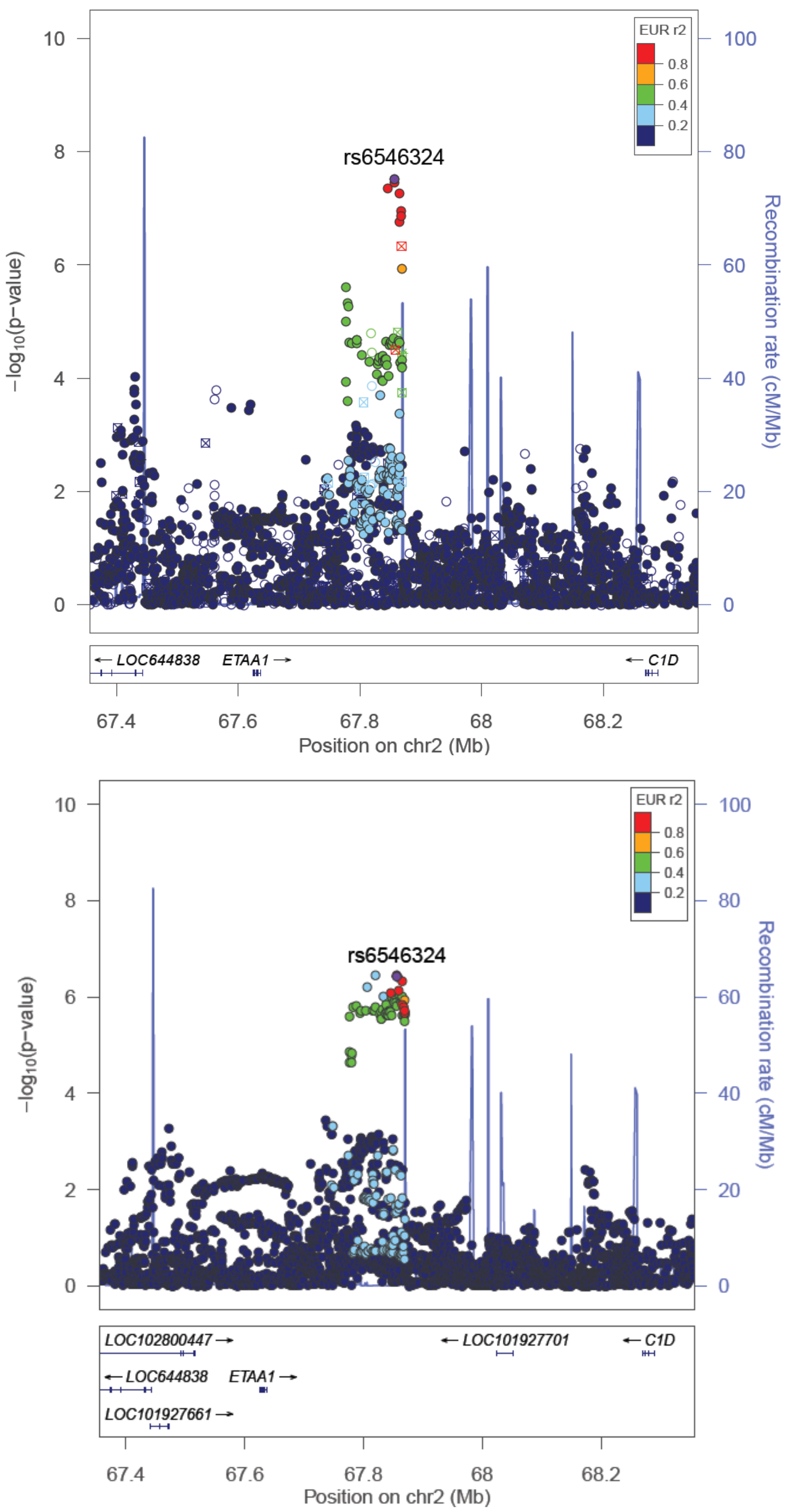

Supplementary Figure 16 | Evidence of association from the fixed-effects GWA meta-analysis including all (top figure) and Grade B (bottom figure) endometriosis cases across the 2p14 region. SNPs are shown as circles, diamonds or squares (filled or unfilled), with the top SNP represented by purple color. All other SNPs are color coded according to the strength of LD with the top SNP (as measured by r^2 in the European 1000 Genomes data).

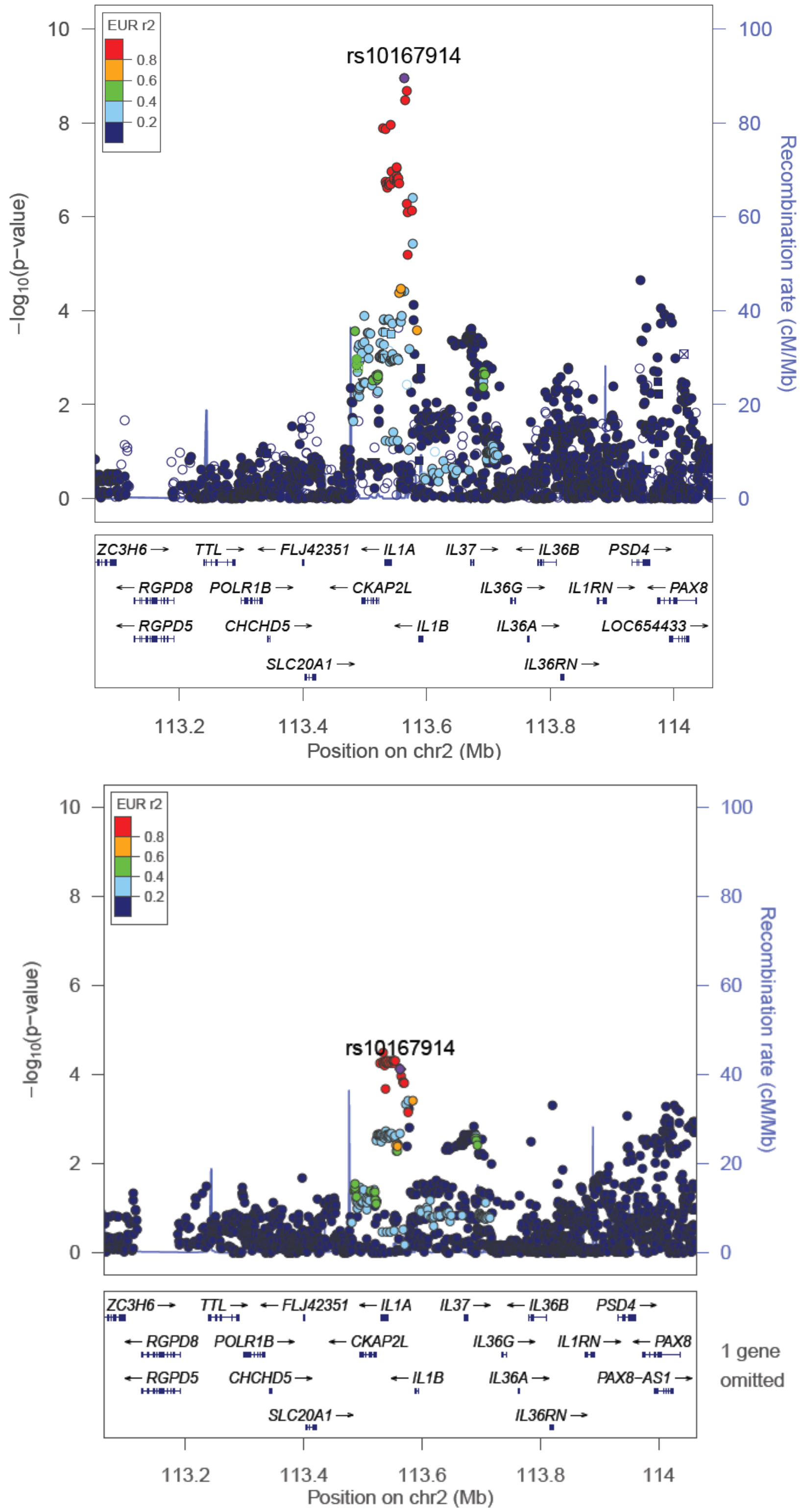

Supplementary Figure 17 | Evidence of association from the fixed-effects GWA meta-analysis including all (top figure) and Grade B (bottom figure) endometriosis cases across the 2q13 region. SNPs are shown as circles, diamonds or squares (filled or unfilled), with the top SNP represented by purple color. All other SNPs are color coded according to the strength of LD with the top SNP (as measured by r^2 in the European 1000 Genomes data).

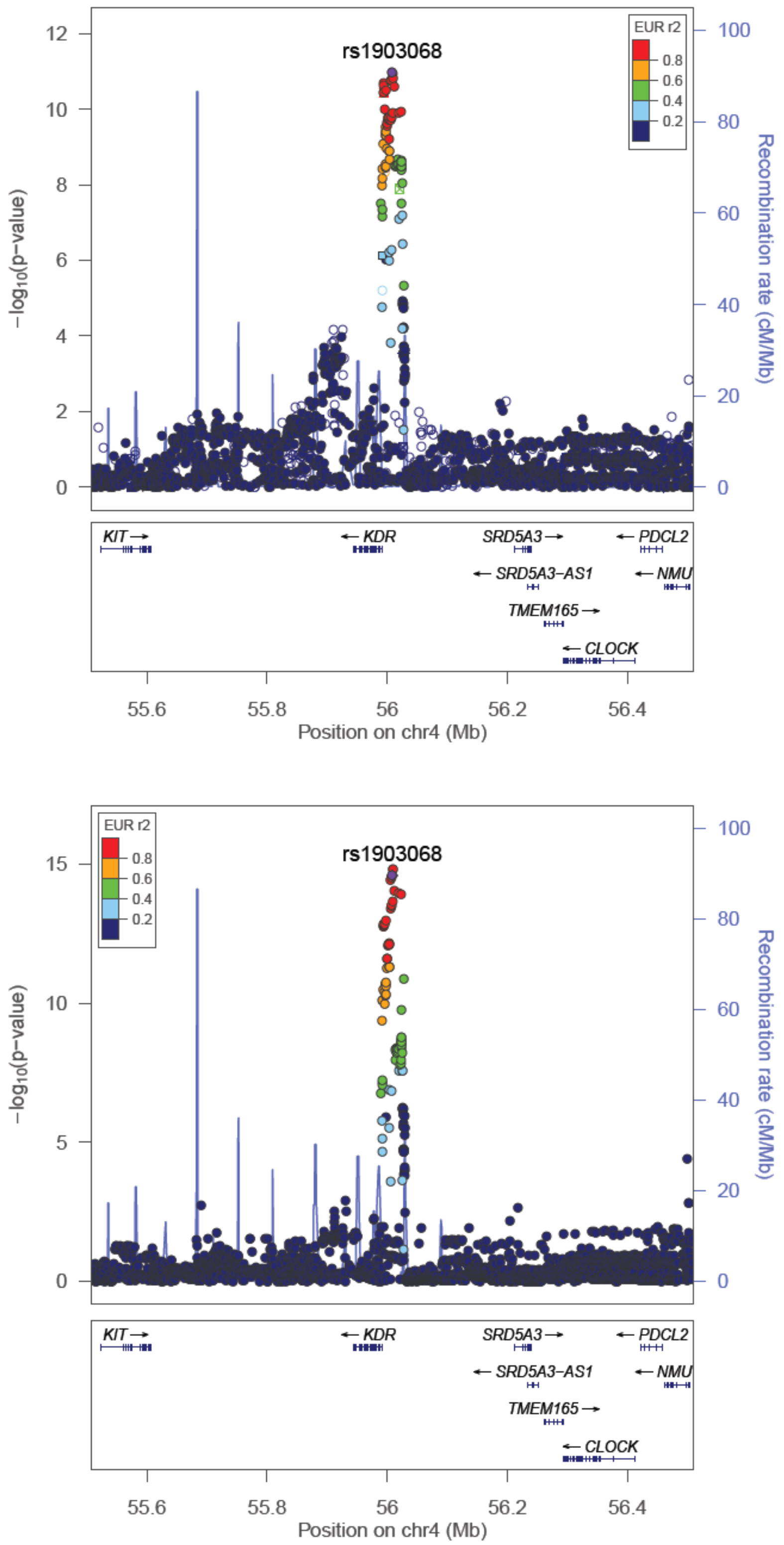

Supplementary Figure 18 | Evidence of association from the fixed-effects GWA meta-analysis including all (top figure) and Grade B (bottom figure) endometriosis cases across the 4q12 region. SNPs are shown as circles, diamonds or squares (filled or unfilled), with the top SNP represented by purple color. All other SNPs are color coded according to the strength of LD with the top SNP (as measured by r^2 in the European 1000 Genomes data).

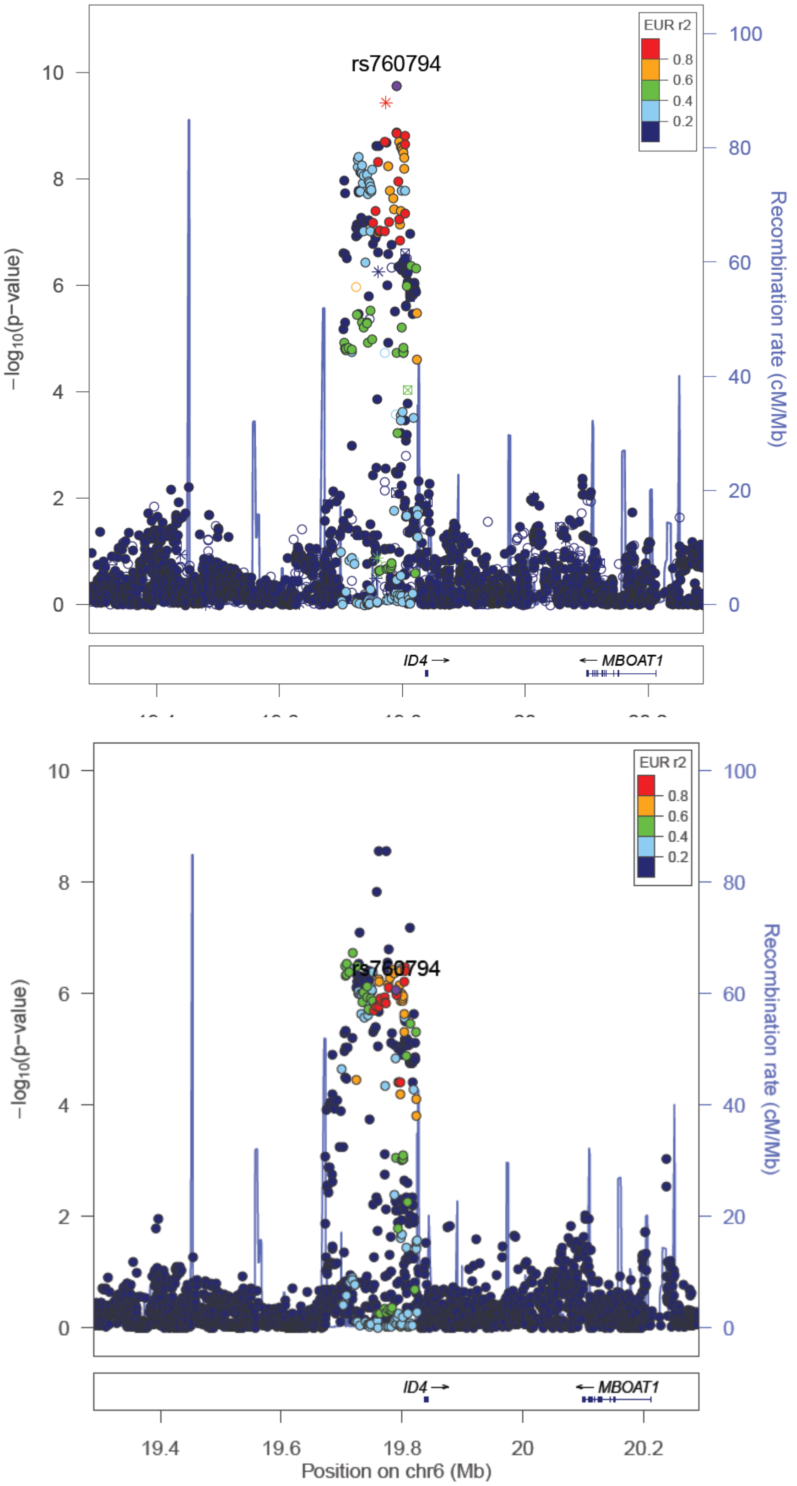

Supplementary Figure 19 | Evidence of association from the fixed-effects GWA meta-analysis including all (top figure) and Grade B (bottom figure) endometriosis cases across the 6p22.3 region. SNPs are shown as circles, diamonds or squares (filled or unfilled), with the top SNP represented by purple color. All other SNPs are color coded according to the strength of LD with the top SNP (as measured by r^2 in the European 1000 Genomes data).

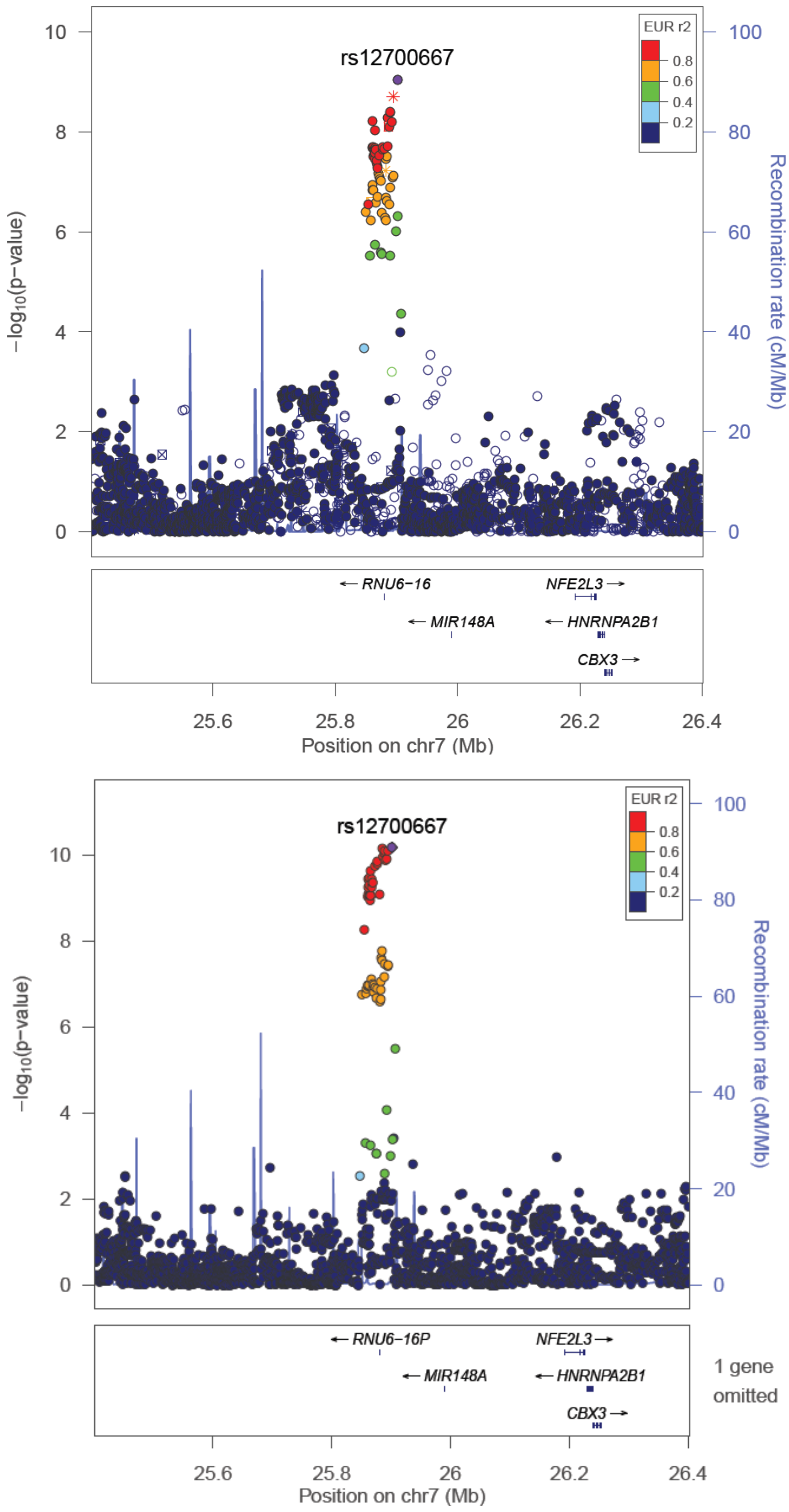

Supplementary Figure 20 | Evidence of association from the fixed-effects GWA meta-analysis including all (top figure) and Grade B (bottom figure) endometriosis cases across the 7p15.2 region. SNPs are shown as circles, diamonds or squares (filled or unfilled), with the top SNP represented by purple color. All other SNPs are color coded according to the strength of LD with the top SNP (as measured by r^2 in the European 1000 Genomes data).

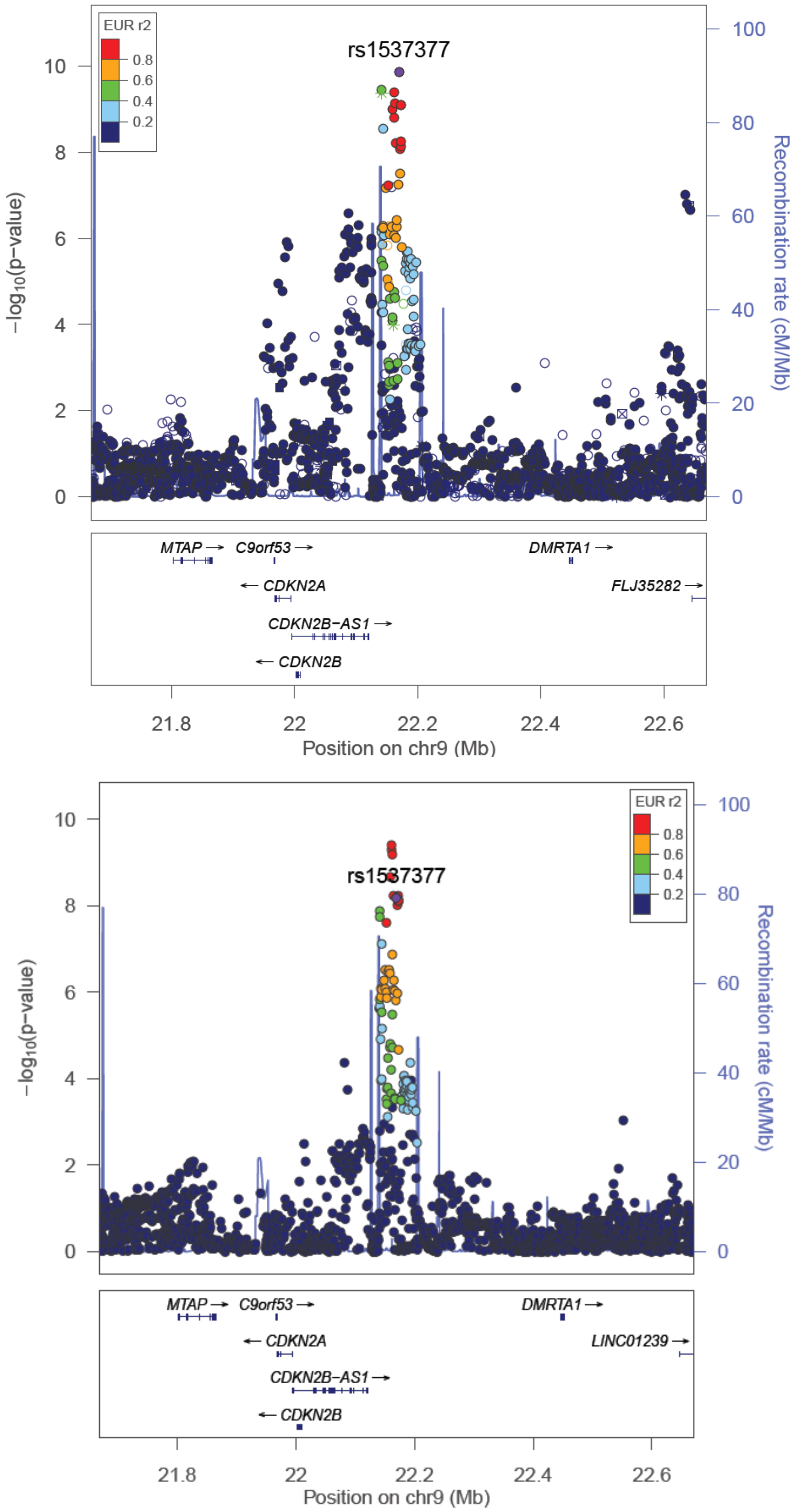

Supplementary Figure 21 | Evidence of association from the fixed-effects GWA meta-analysis including all (top figure) and Grade B (bottom figure) endometriosis cases across the 9p21.3 region. SNPs are shown as circles, diamonds or squares (filled or unfilled), with the top SNP represented by purple color. All other SNPs are color coded according to the strength of LD with the top SNP (as measured by r^2 in the European 1000 Genomes data).

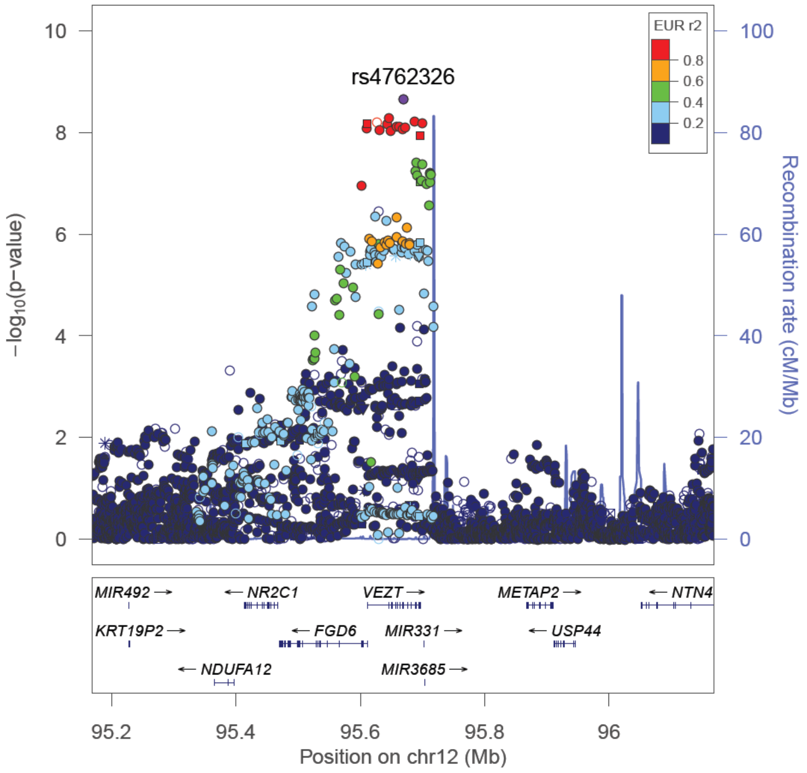

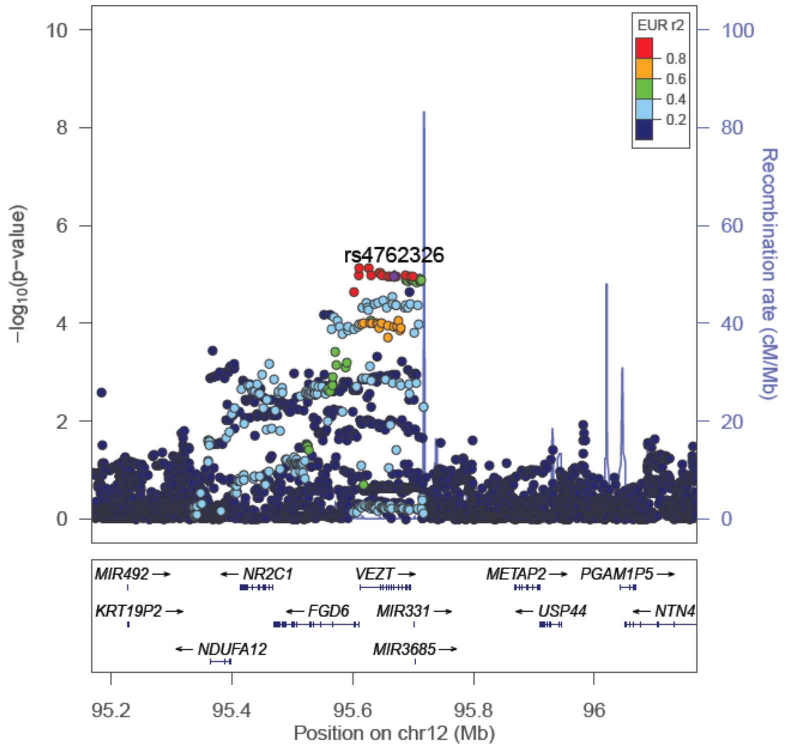

Supplementary Figure 22 | Evidence of association from the fixed-effects GWA meta-analysis including all (top figure) and Grade B (bottom figure) endometriosis cases across the 12q22 region. SNPs are shown as circles, diamonds or squares (filled or unfilled), with the top SNP represented by purple color. All other SNPs are color coded according to the strength of LD with the top SNP (as measured by r^2 in the European 1000 Genomes data).

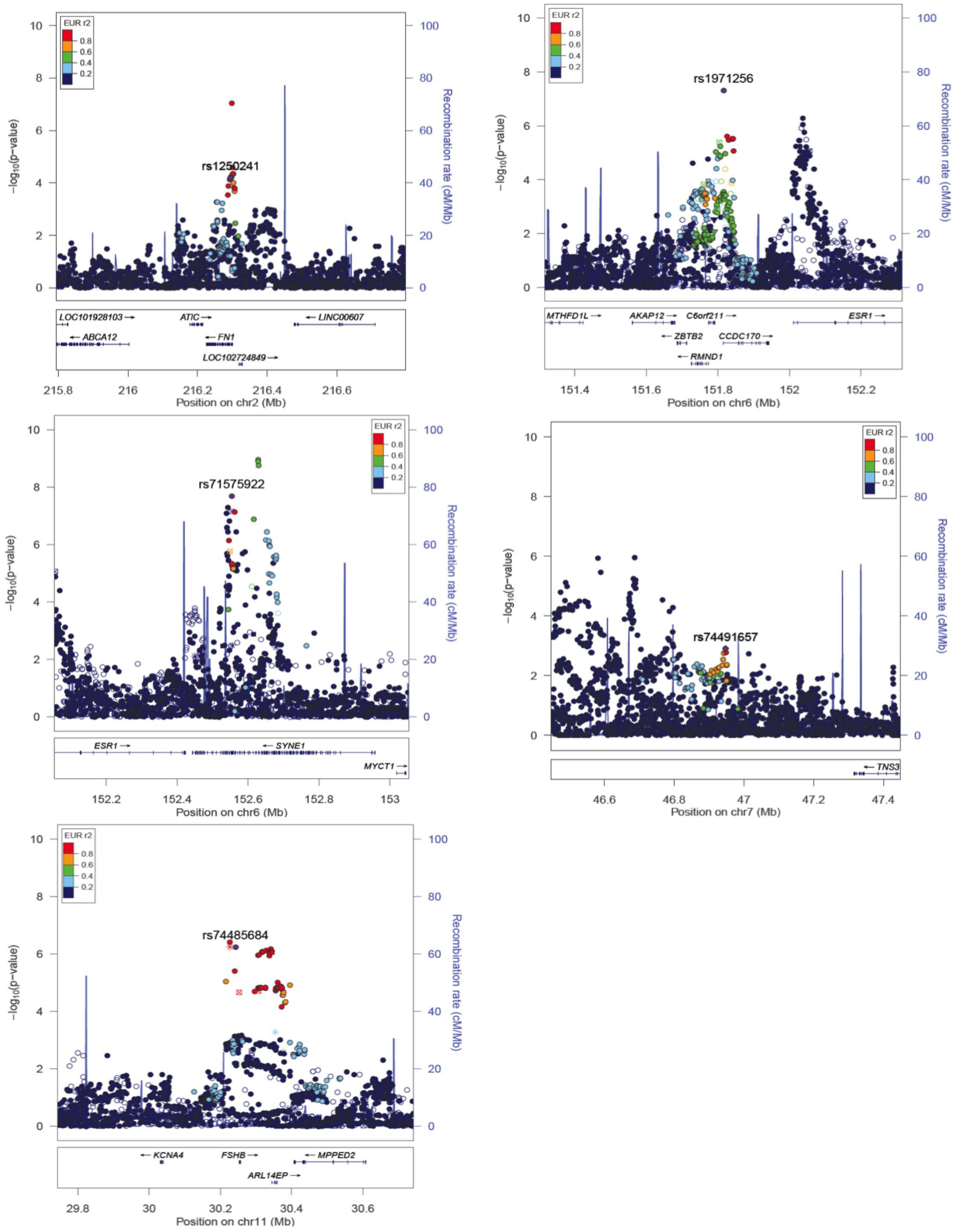

Supplementary Figure 23 | Evidence of association from the fixed-effects GWA meta-analysis including all endometriosis cases across the 2q35 and 7p12.3 regions, and including Grade B cases across the 6q25.1 (*CDC170* and *SYNE1*), and 11p14.1 regions. SNPs are shown as circles, diamonds or squares (filled or unfilled), with the top SNP represented by purple color. All other SNPs are color coded according to the strength of LD with the top SNP (as measured by r^2 in the European 1000 Genomes data).

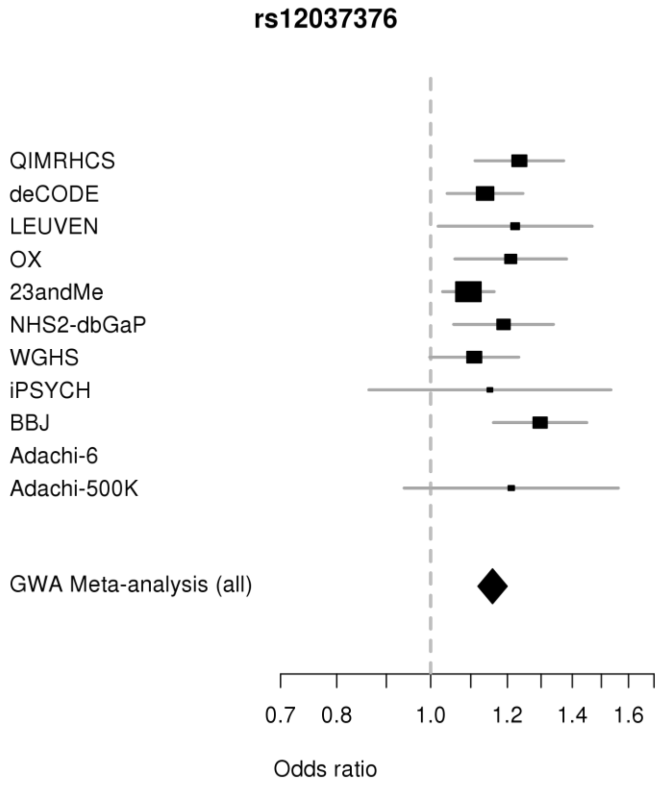

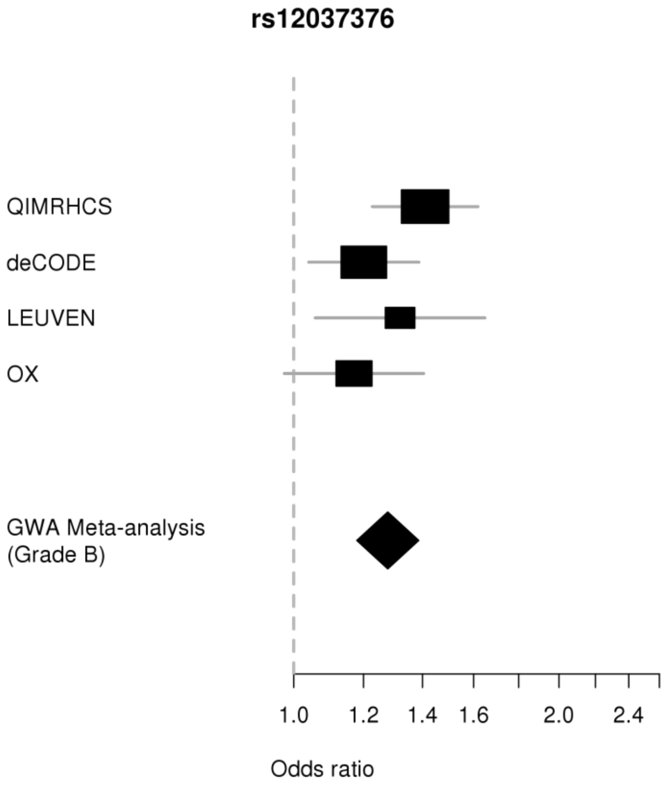

Supplementary Figure 24 | Forest plots of risk allele effects for top SNP (rs12037376) at 1p36.12 locus in the individual case-control datasets and GWA meta-analysis including all (left) and Grade B (right) endometriosis cases.

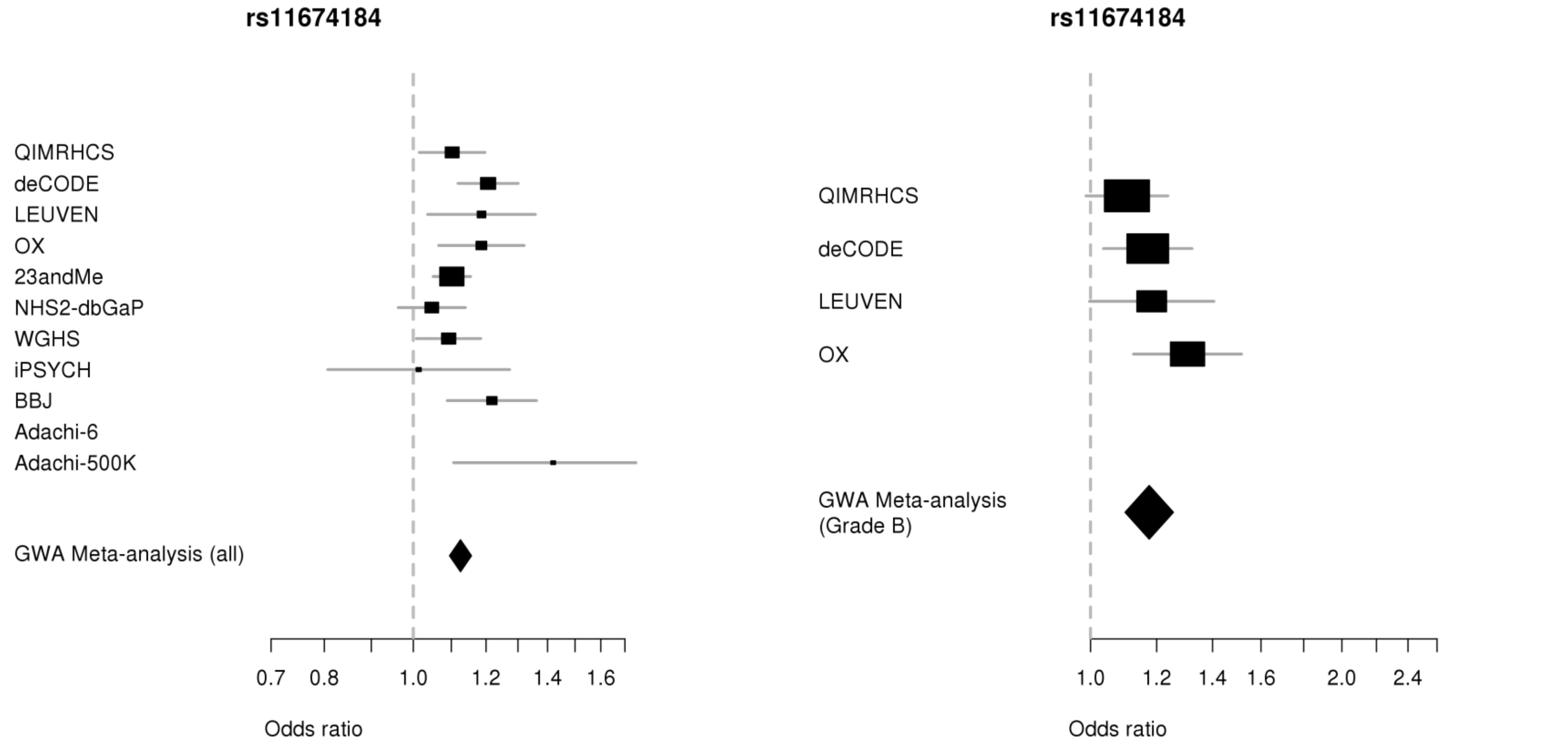

Supplementary Figure 25 | Forest plots of risk allele effects for top SNP (rs11674184) at 2p25.1 locus in the individual case-control datasets and GWA meta-analysis including all (left) and Grade B (right) endometriosis cases.

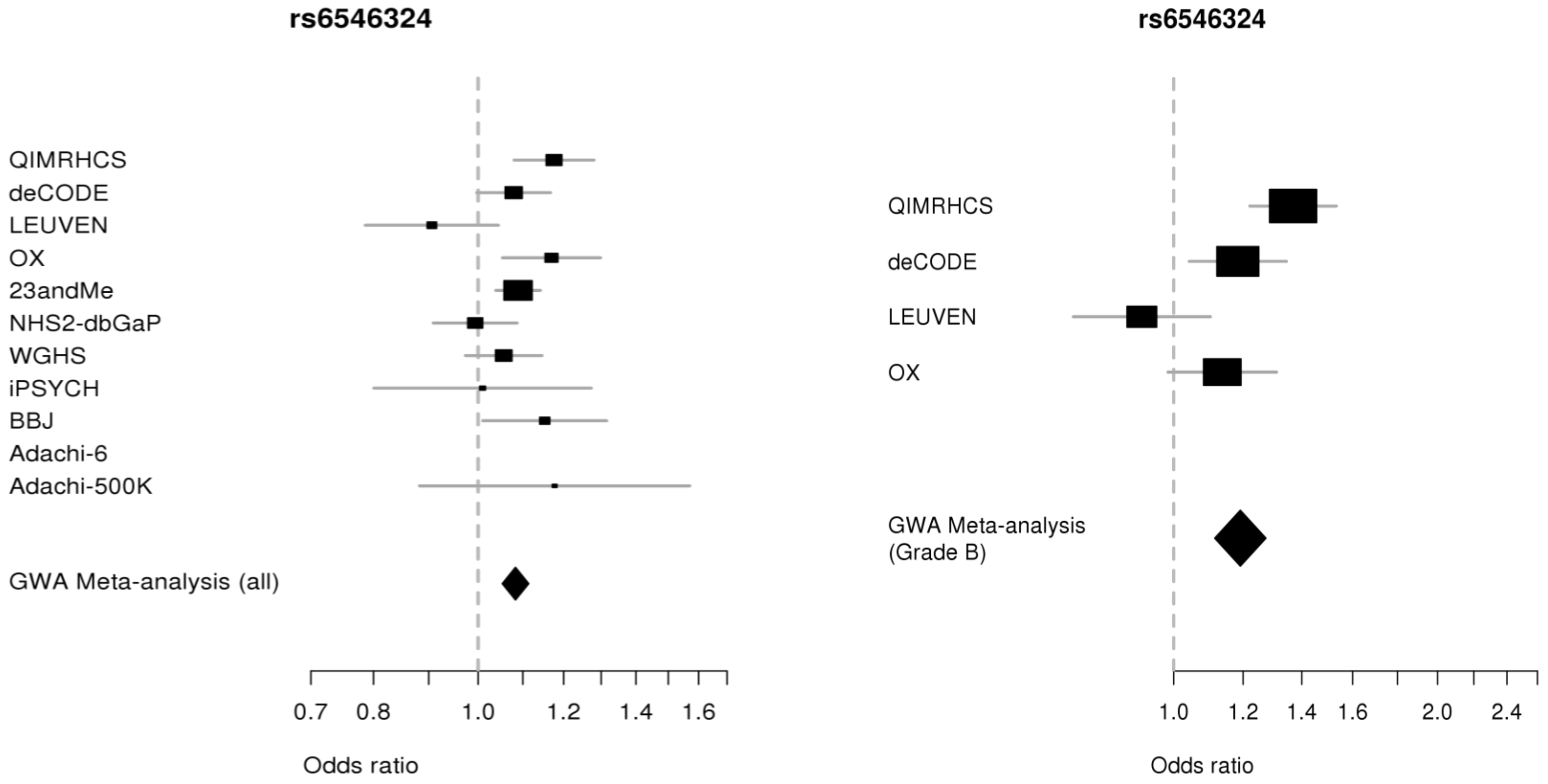

Supplementary Figure 26 | Forest plots of risk allele effects for top SNP (rs6546324) at 2p14 locus in the individual case-control datasets and GWA meta-analysis including all (left) and Grade B (right) endometriosis cases.

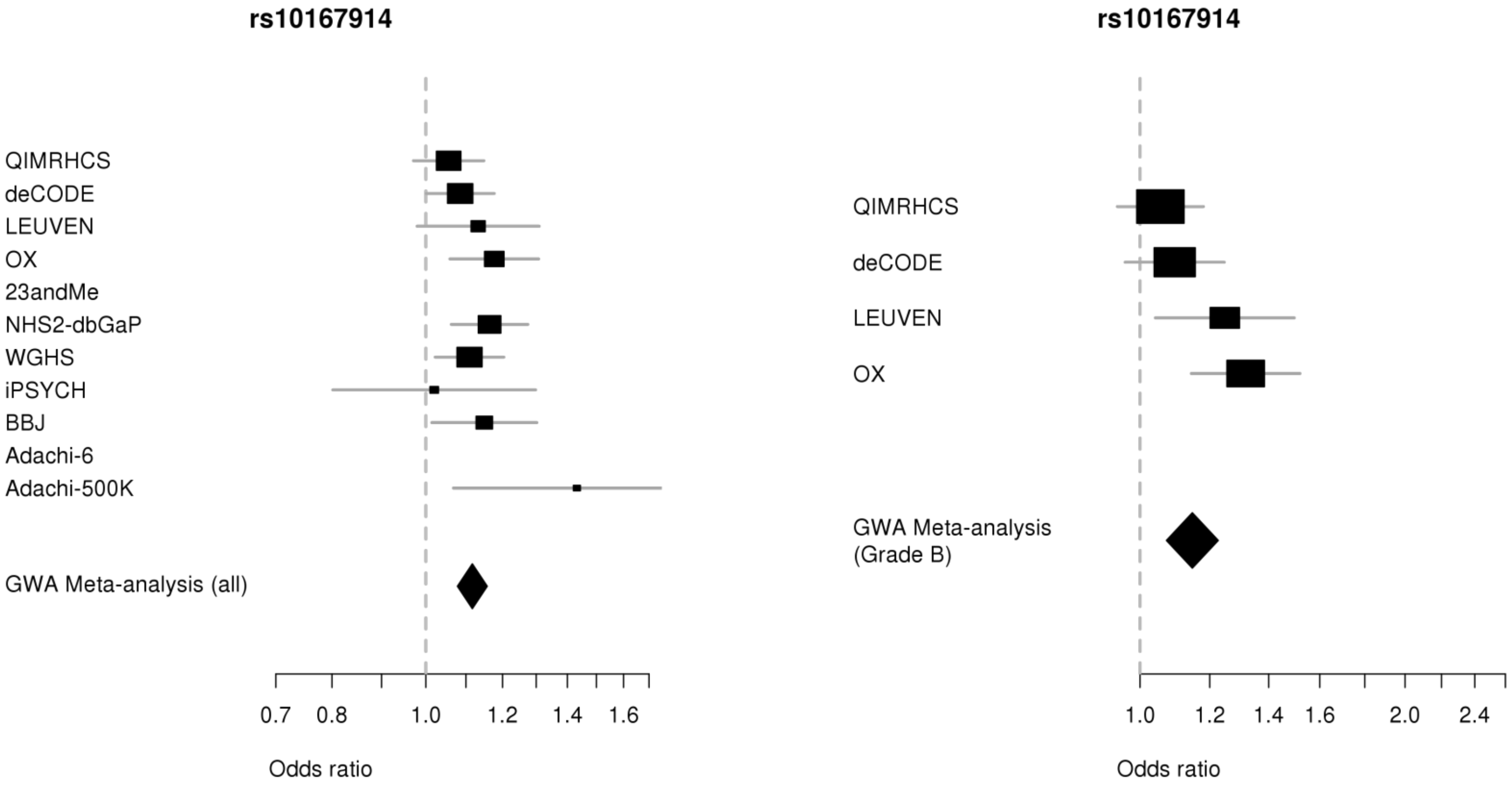

Supplementary Figure 27 | Forest plots of risk allele effects for top SNP (rs10167914) at 2q13 locus in the individual case-control datasets and GWA meta-analysis including all (left) and Grade B (right) endometriosis cases.

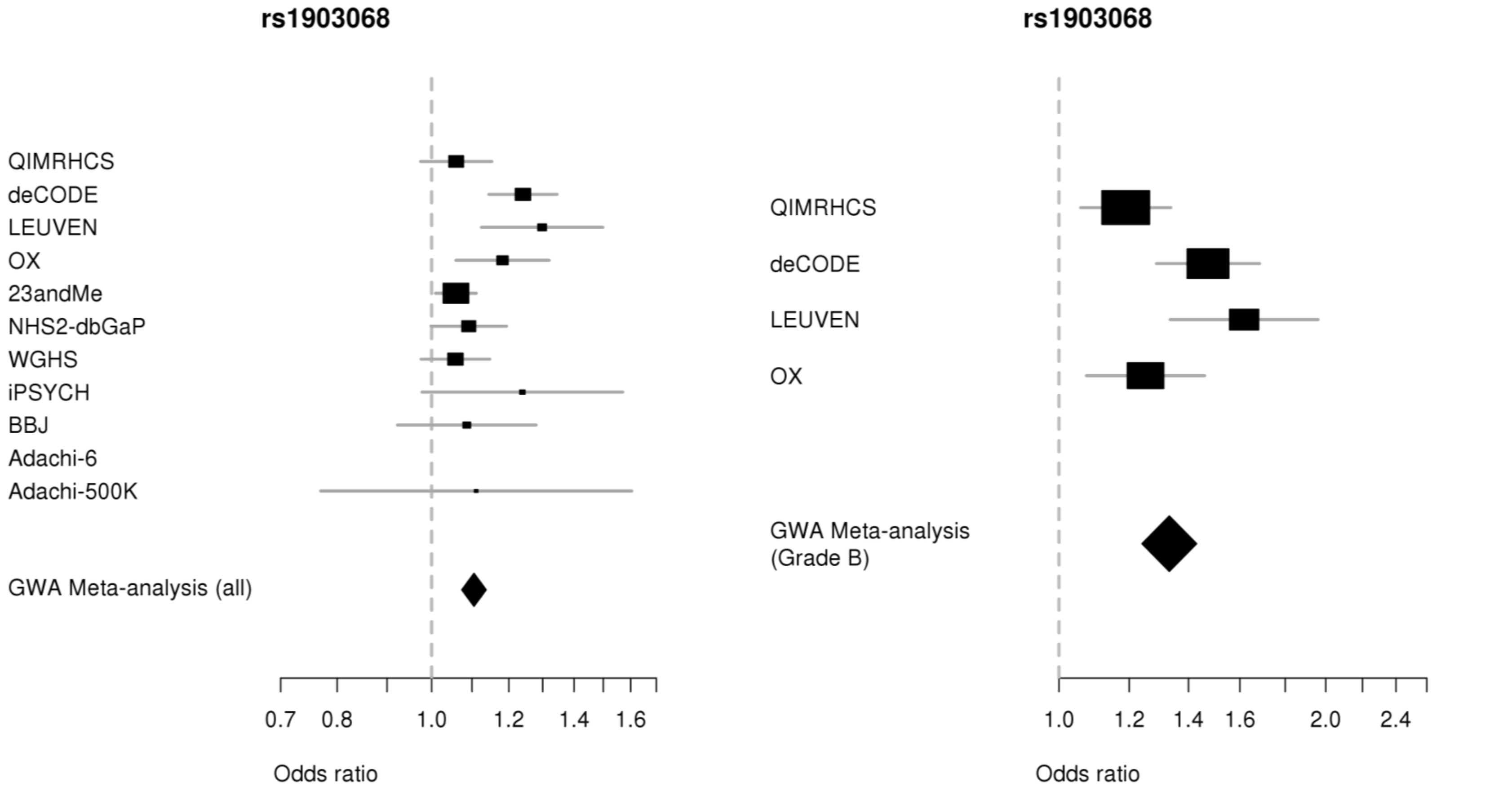

Supplementary Figure 28 | Forest plots of risk allele effects for top SNP (rs1903068) at 4q12 locus in the individual case-control datasets and GWA meta-analysis including all (left) and Grade B (right) endometriosis cases.

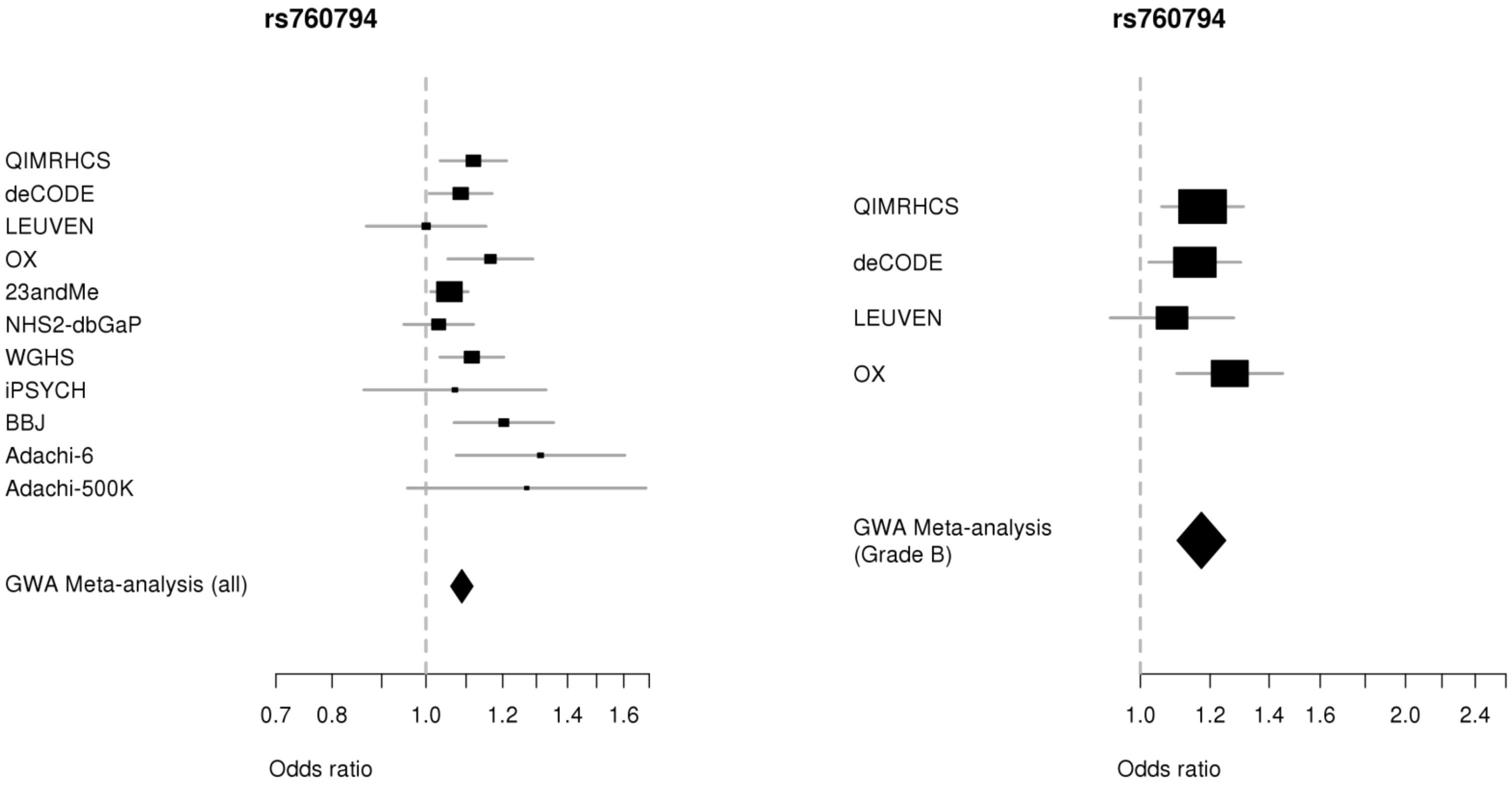

Supplementary Figure 29 | Forest plots of risk allele effects for top SNP (rs760794) at 6p22.3 locus in the individual case-control datasets and GWA meta-analysis including all (left) and Grade B (right) endometriosis cases.

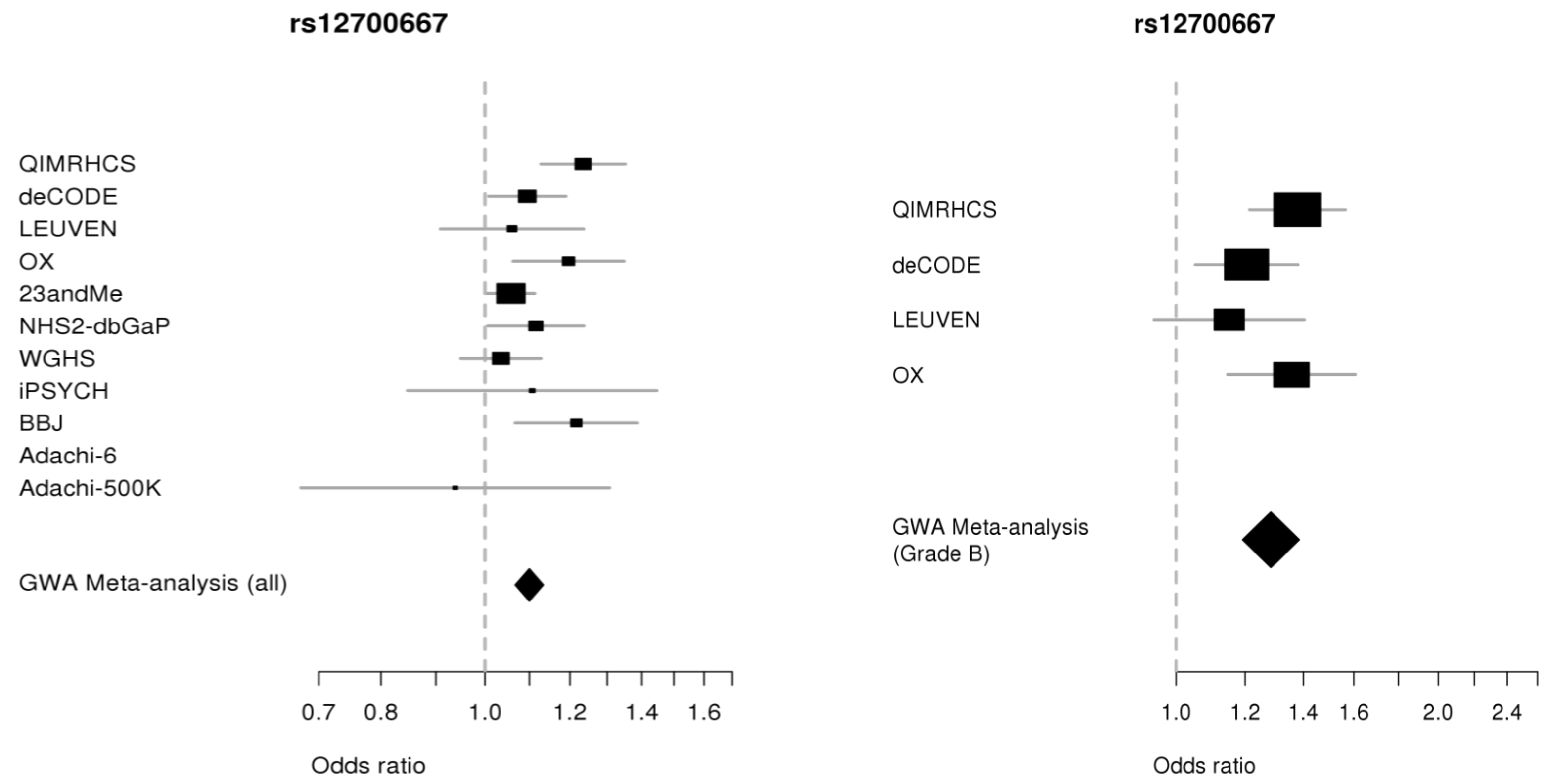

Supplementary Figure 30 | Forest plots of risk allele effects for top SNP (rs12700667) at 7p15.2 locus in the individual case-control datasets and GWA meta-analysis including all (left) and Grade B (right) endometriosis cases.

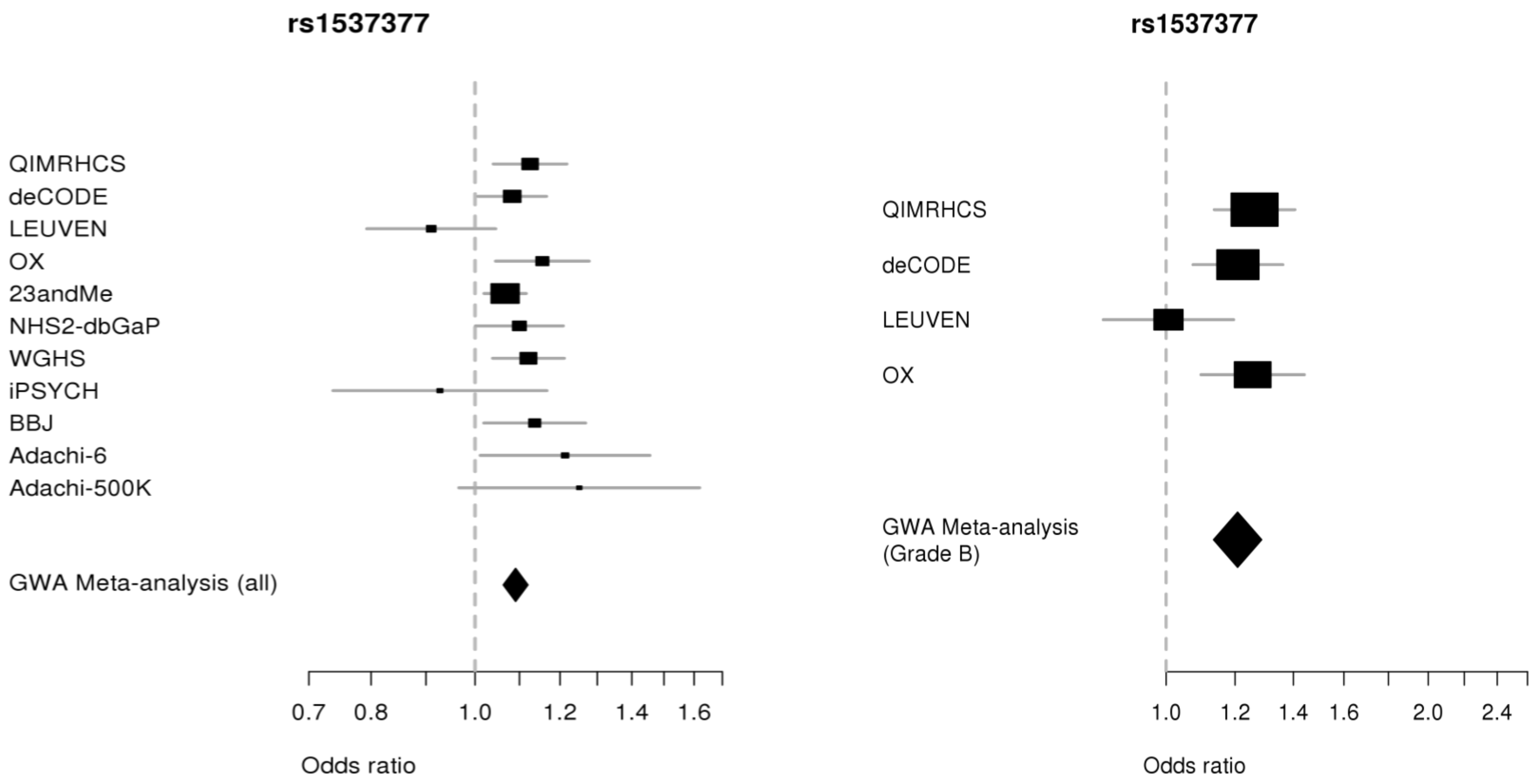

Supplementary Figure 31 | Forest plots of risk allele effects for top SNP (rs1537377) at 9p21.3 locus in the individual case-control datasets and GWA meta-analysis including all (left) and Grade B (right) endometriosis cases.

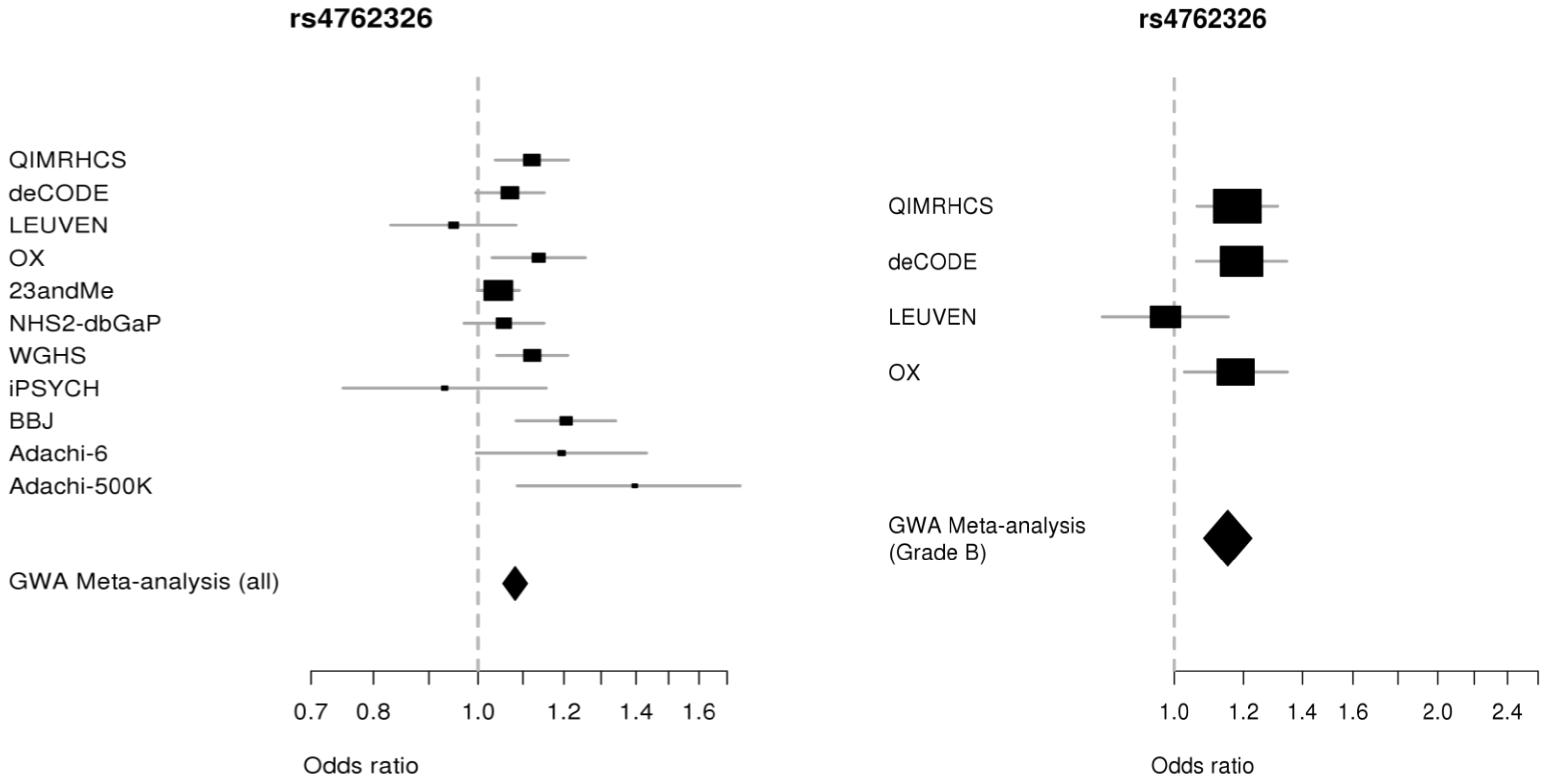

Supplementary Figure 32 | Forest plots of risk allele effects for top SNP (rs4762326) at 12q22 locus in the individual case-control datasets and GWA meta-analysis including all (left) and Grade B (right) endometriosis cases.

rs1250241

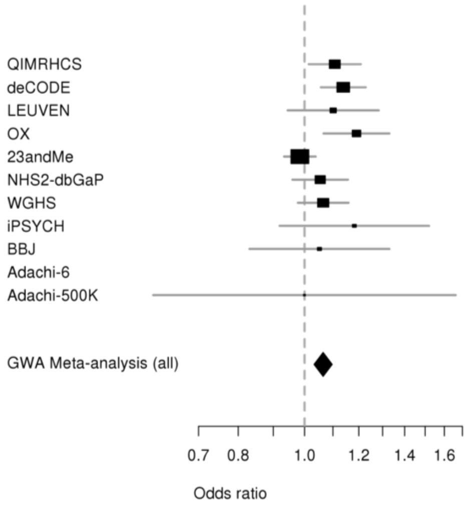

rs1971256

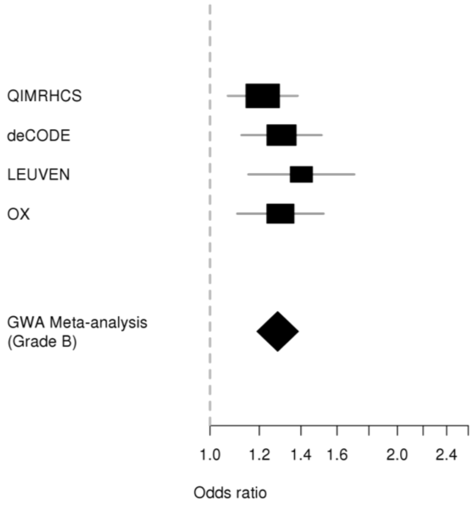

rs71575922

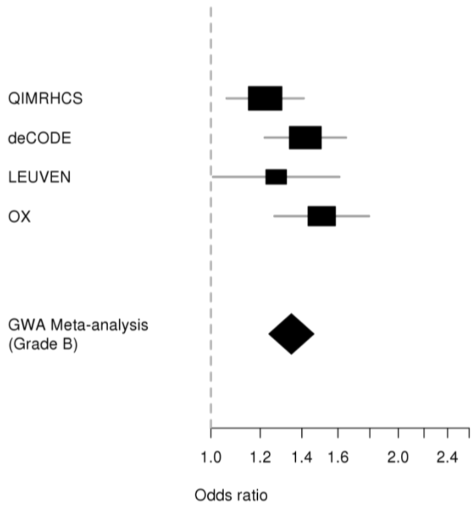

rs74491657

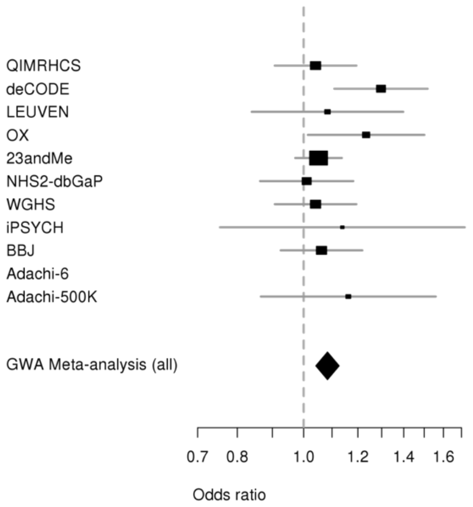

rs74485684

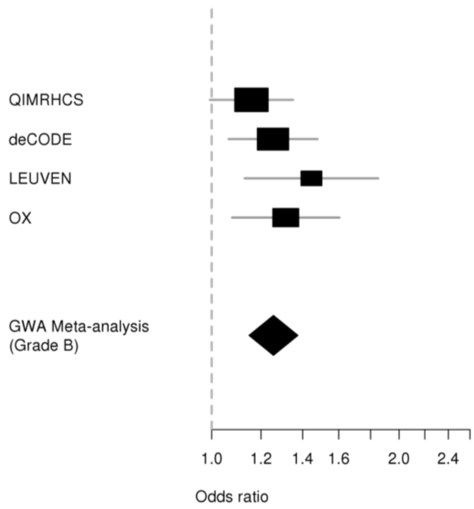

Supplementary Figure 33 | Forest plots of risk allele effects for the 5 novel SNPs (rs1250241, rs1971256, rs71575922, rs74491657 and rs74485684) at 2q35, 6q25.1 (*CCDC170* and *SYNE1*), 7p12.3 and 11p14.1 in the individual case-control datasets and GWA meta-analysis including all (for rs1250241 and rs74491657) and Grade B (for rs1971256, rs71575922 and rs74485684) cases.

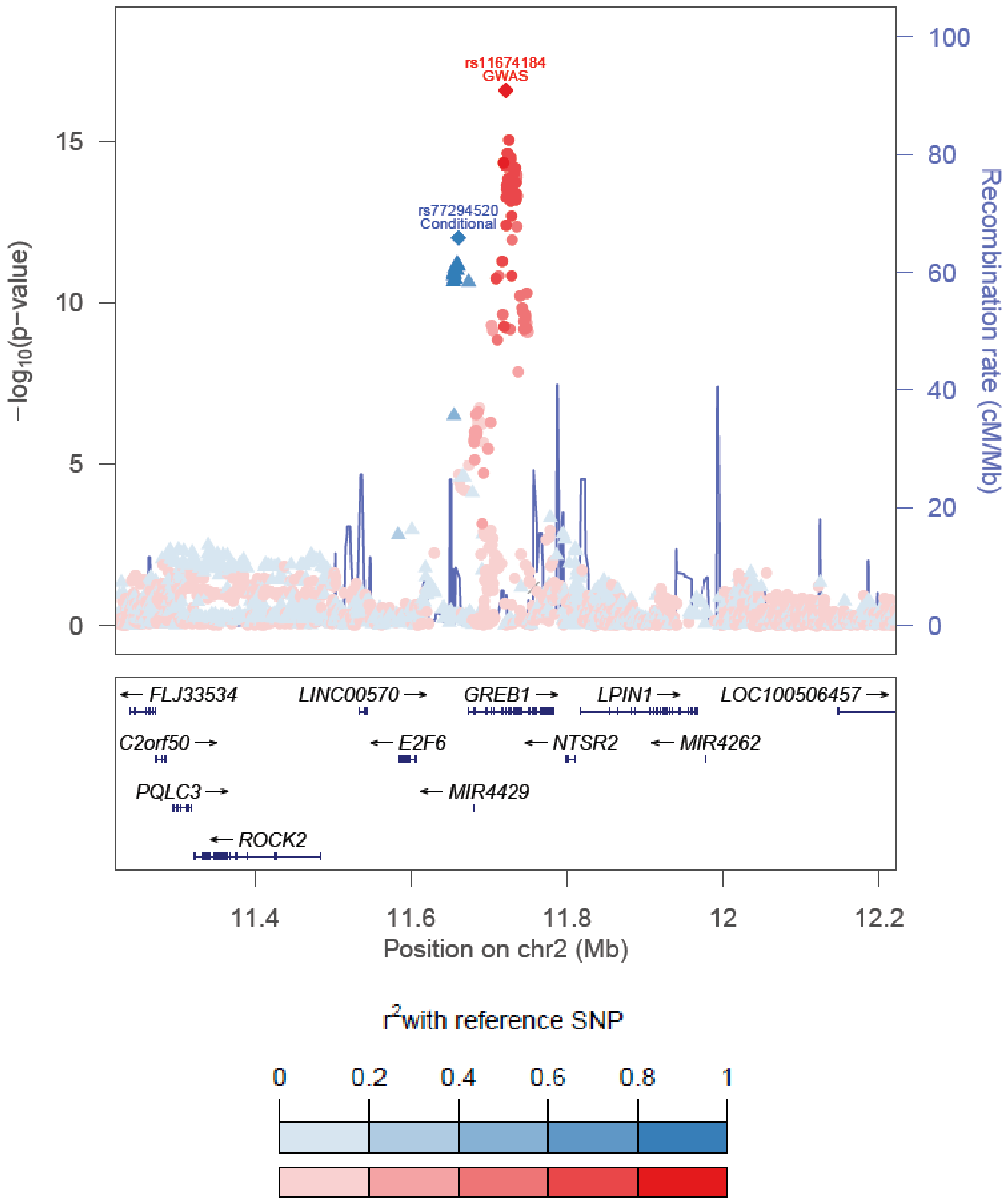

Supplementary Figure 34 | Evidence of association from the GWA meta-analysis including all endometriosis cases across the 2p25.1 region, with secondary association signal identified by GCTA conditional analysis. Primary top SNP and the secondary top SNP identified from a conditional analysis are represented as red and blue diamonds, respectively. All other SNPs are color coded to match which of the top SNPs it is in highest LD with (as measured by r^2 in the European 1000 Genomes data). The extent of LD with the top SNPs is shown by a gradient of color.

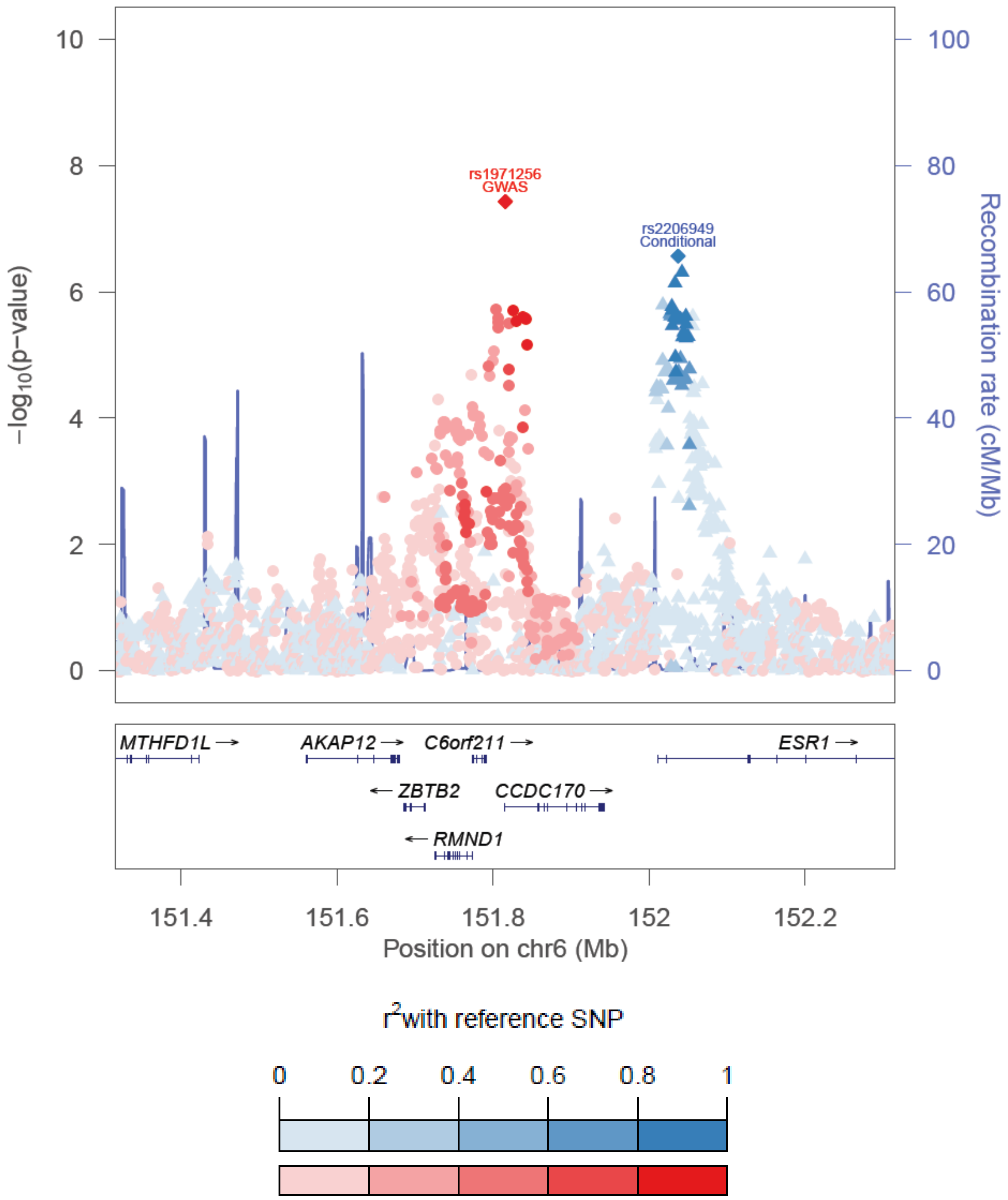

Supplementary Figure 35 | Evidence of association from the GWA meta-analysis including all endometriosis cases across the 6q25.1 (*CDC170*) region, with secondary association signal identified by GCTA conditional analysis. Primary top SNP and the secondary top SNP identified from a conditional analysis are represented as red and blue diamonds, respectively. All other SNPs are color coded to match which of the top SNPs it is in highest LD with (as measured by r^2 in the European 1000 Genomes data). The extent of LD with the top SNPs is shown by a gradient of color.

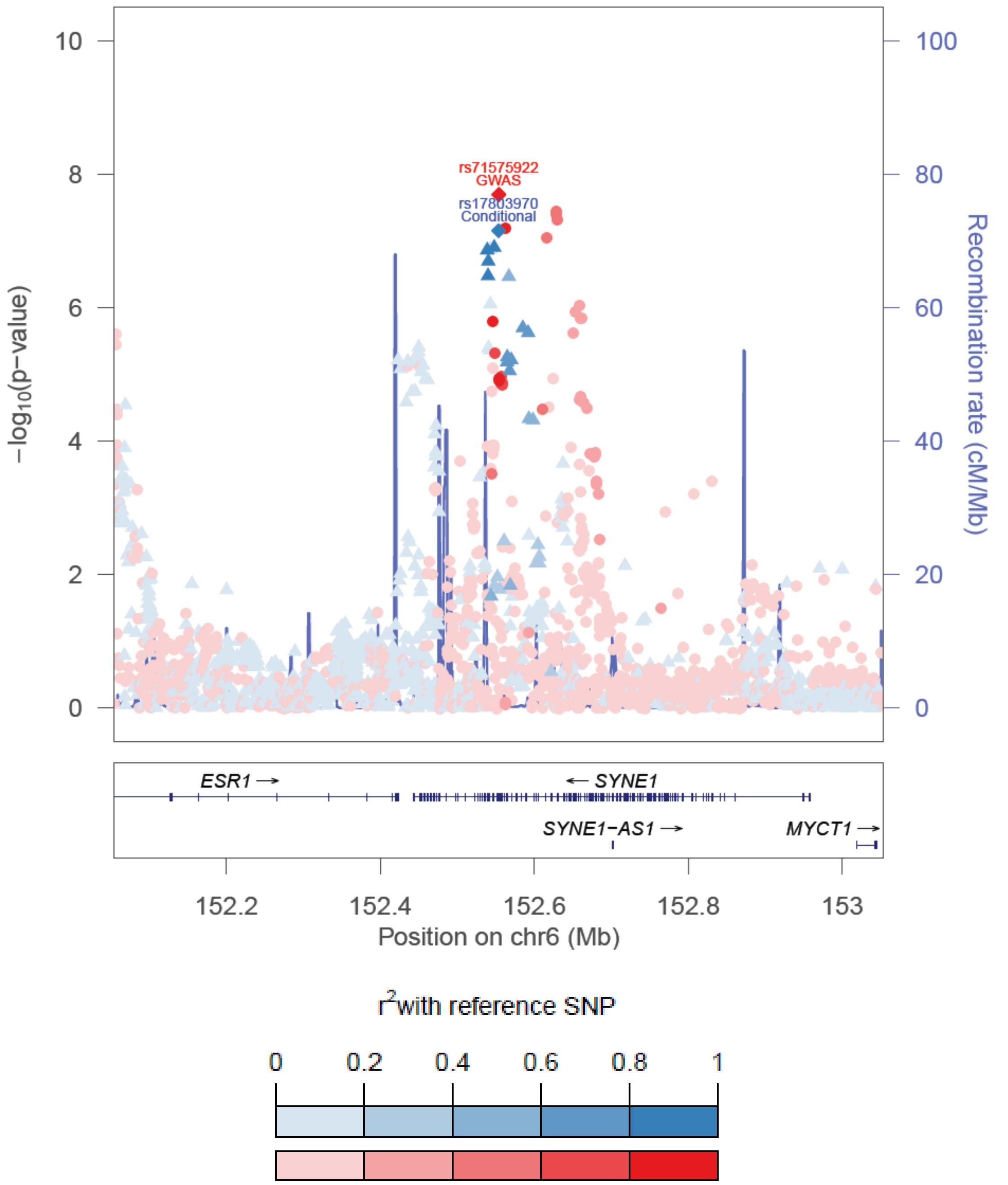

Supplementary Figure 36 | Evidence of association from the GWA meta-analysis including all endometriosis cases across the 6q25.1 (*SYNE1*) region, with secondary association signal identified by GCTA conditional analysis. Primary top SNP and the secondary top SNP identified from a conditional analysis are represented as red and blue diamonds, respectively. All other SNPs are color coded to match which of the top SNPs it is in highest LD with (as measured by r^2 in the European 1000 Genomes data). The extent of LD with the top SNPs is shown by a gradient of color.

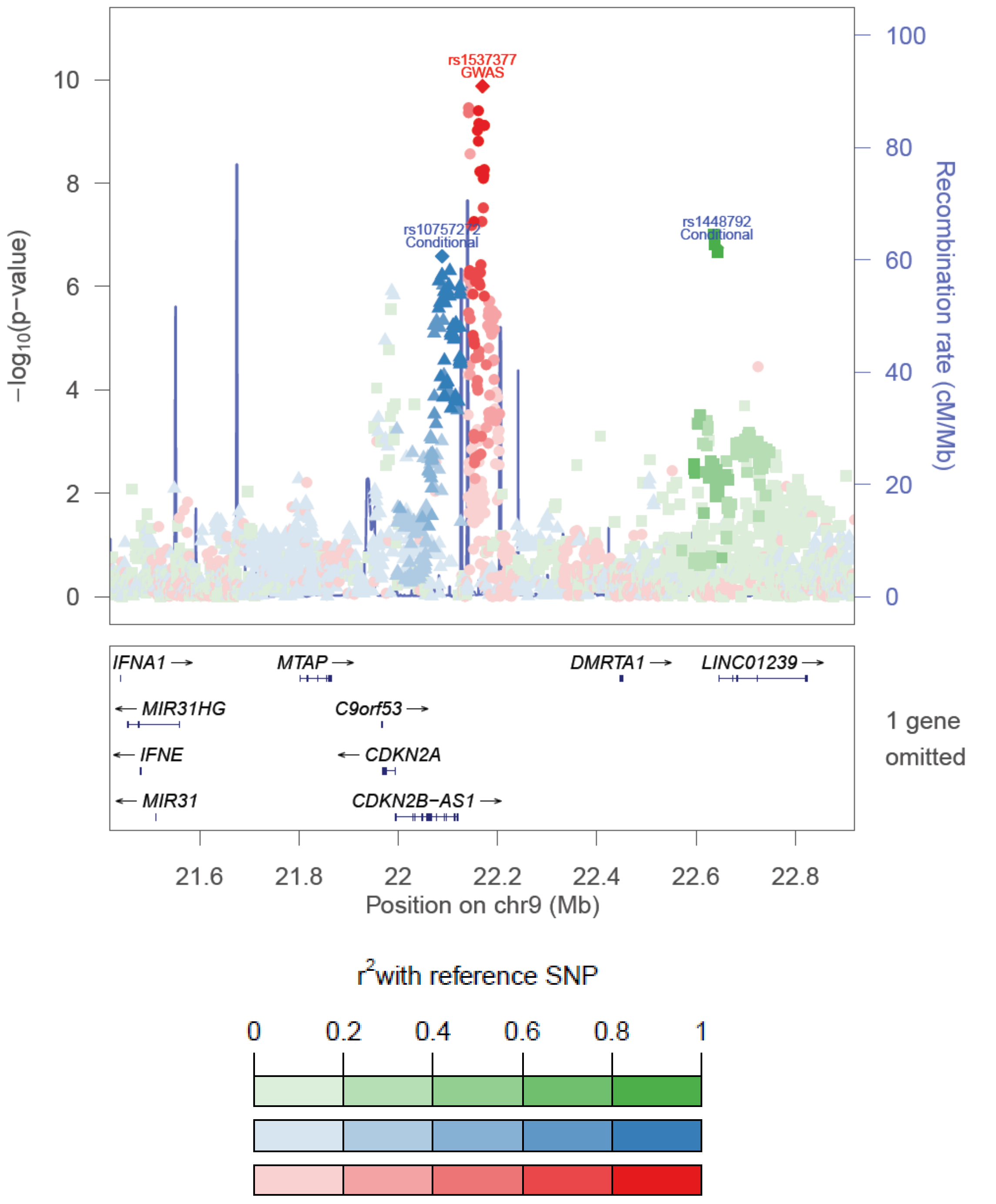

Supplementary Figure 37 | Evidence of association from the GWA meta-analysis including all endometriosis cases across the 9p21.3 region, with secondary association signal identified by GCTA conditional analysis. Primary top SNP and the secondary top SNP identified from a conditional analysis are represented as red and blue diamonds, respectively. All other SNPs are color coded to match which of the top SNPs it is in highest LD with (as measured by r^2 in the European 1000 Genomes data). The extent of LD with the top SNPs is shown by a gradient of color.

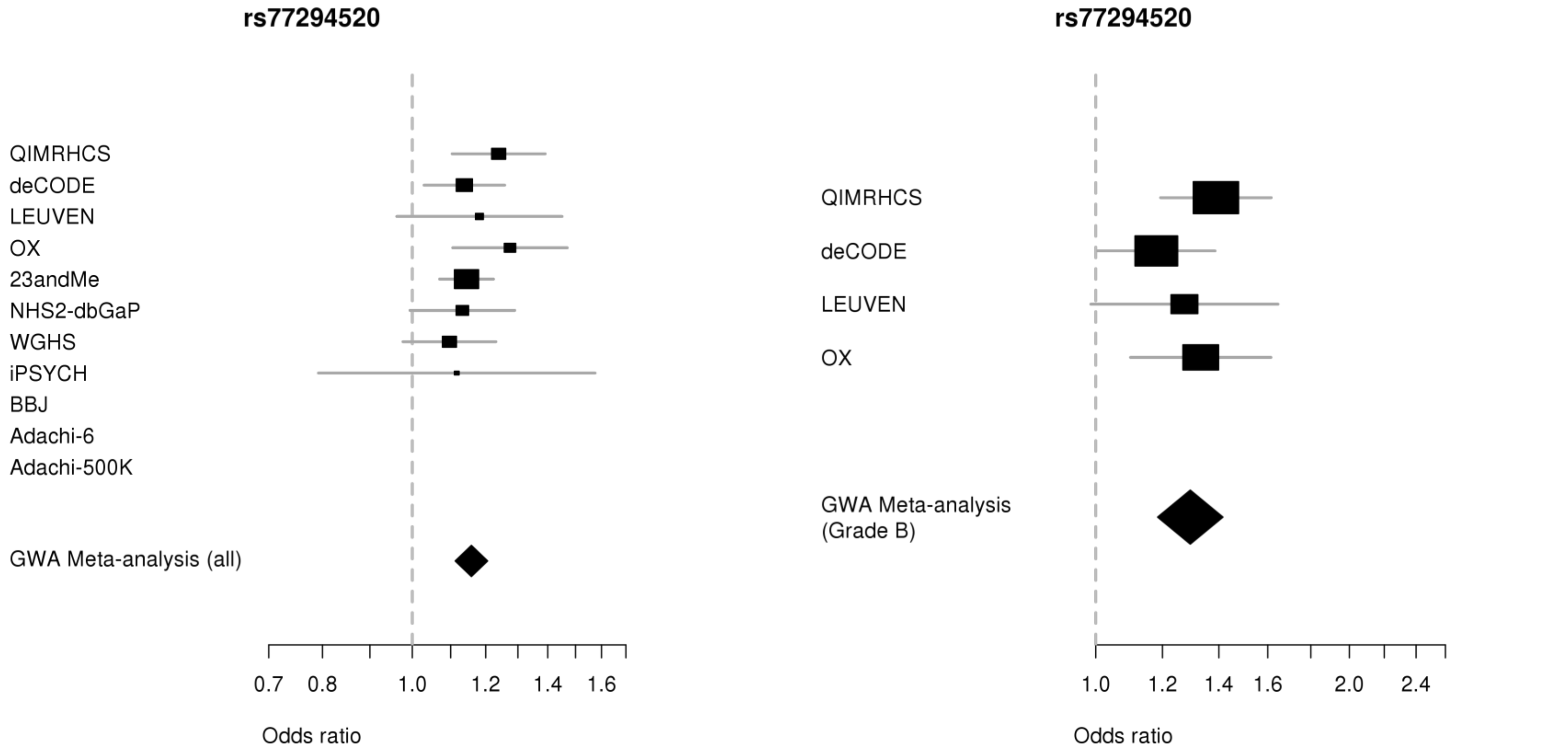

Supplementary Figure 38 | Forest plots of risk allele effects for a secondary association signal at 2p25.1 locus in the individual case-control datasets and GWA meta-analysis including all (left) and Grade B (right) endometriosis cases.

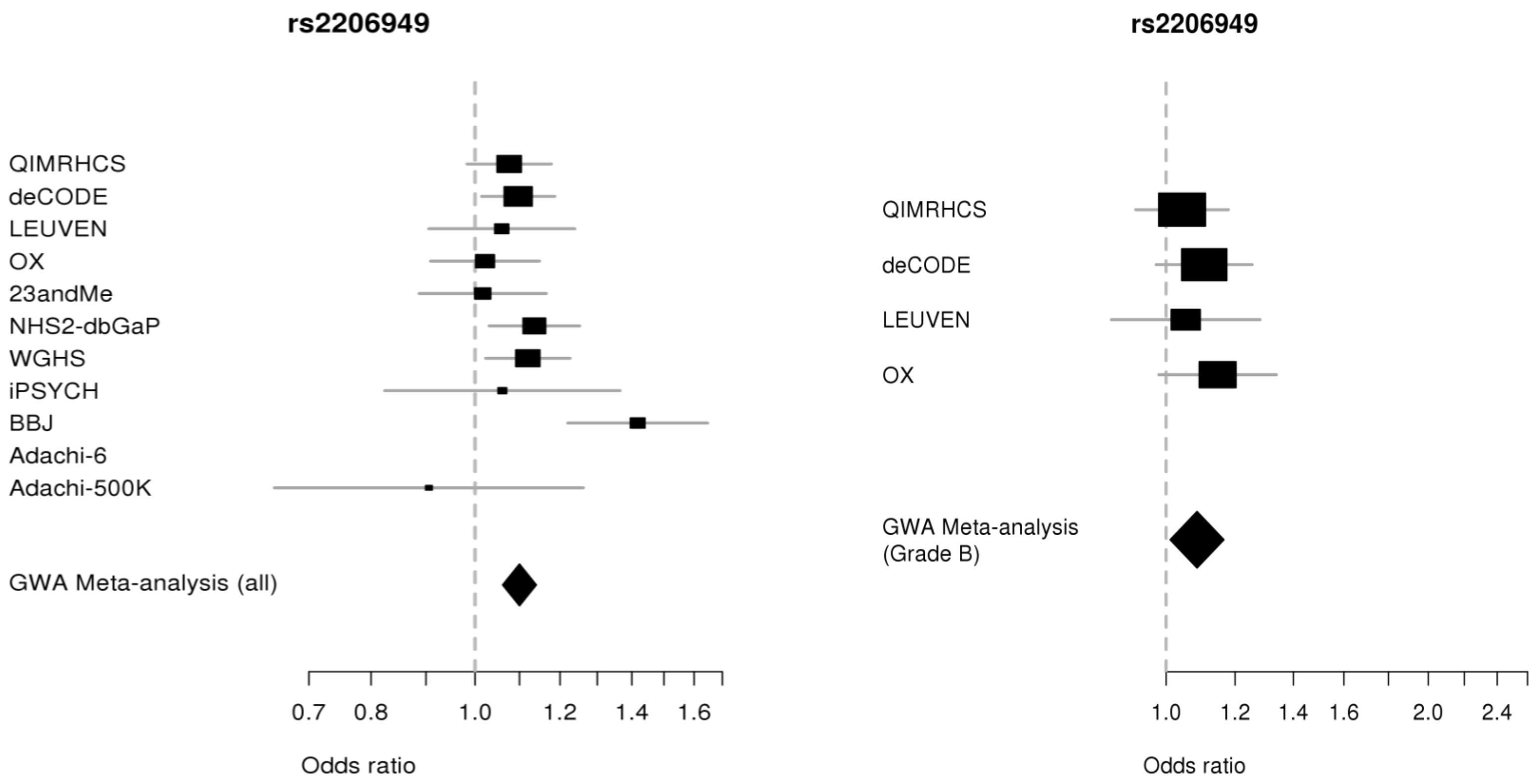

Supplementary Figure 39 | Forest plots of risk allele effects for a secondary association signal at 6q25.1 (*CDC170*) locus in the individual case-control datasets and GWA meta-analysis including all (left) and Grade B (right) endometriosis cases.

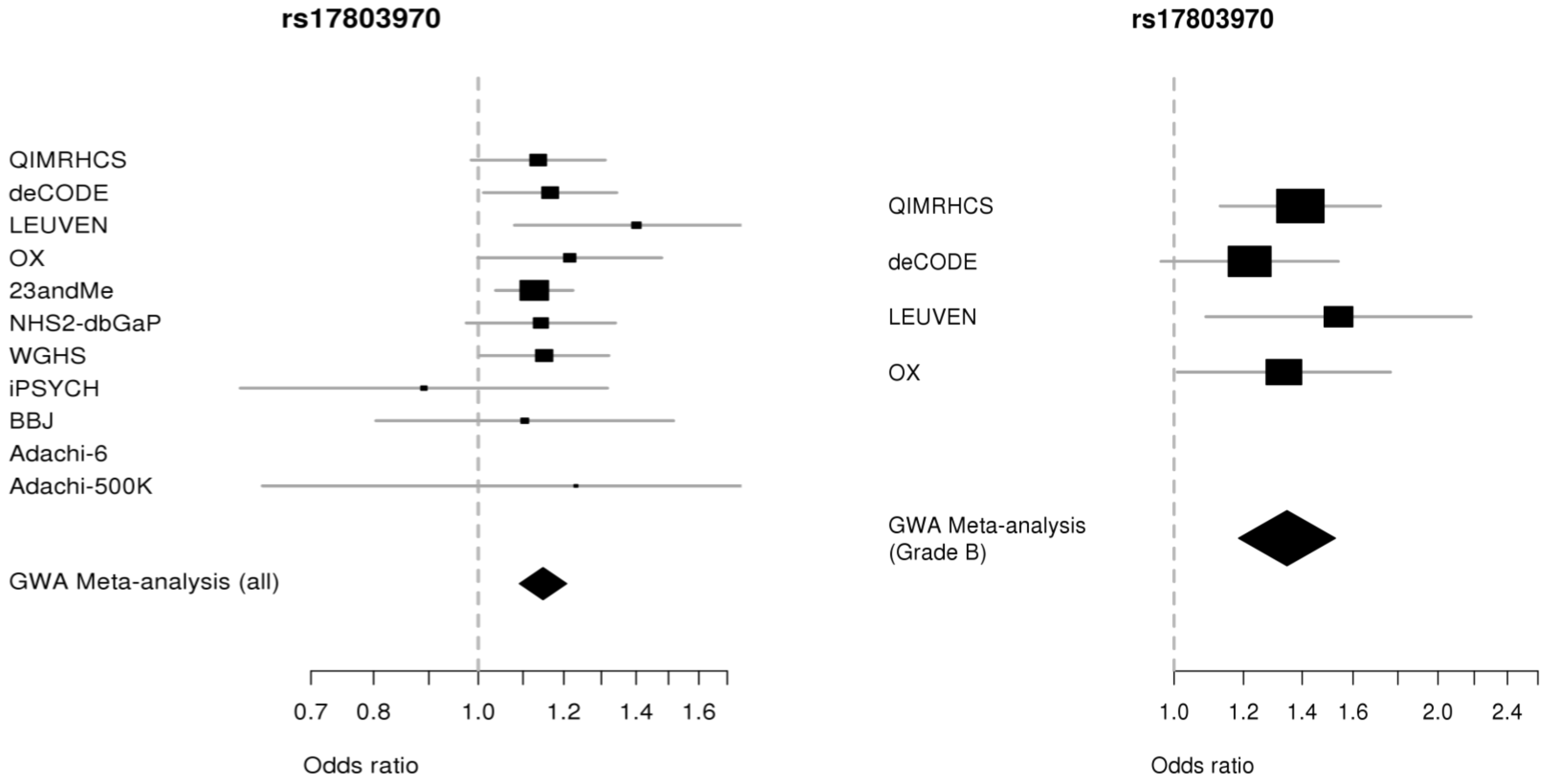

Supplementary Figure 40 | Forest plots of risk allele effects for a secondary association signal at 6q25.1 (*SYNE1*) locus in the individual case-control datasets and GWA meta-analysis including all (left) and Grade B (right) endometriosis cases.

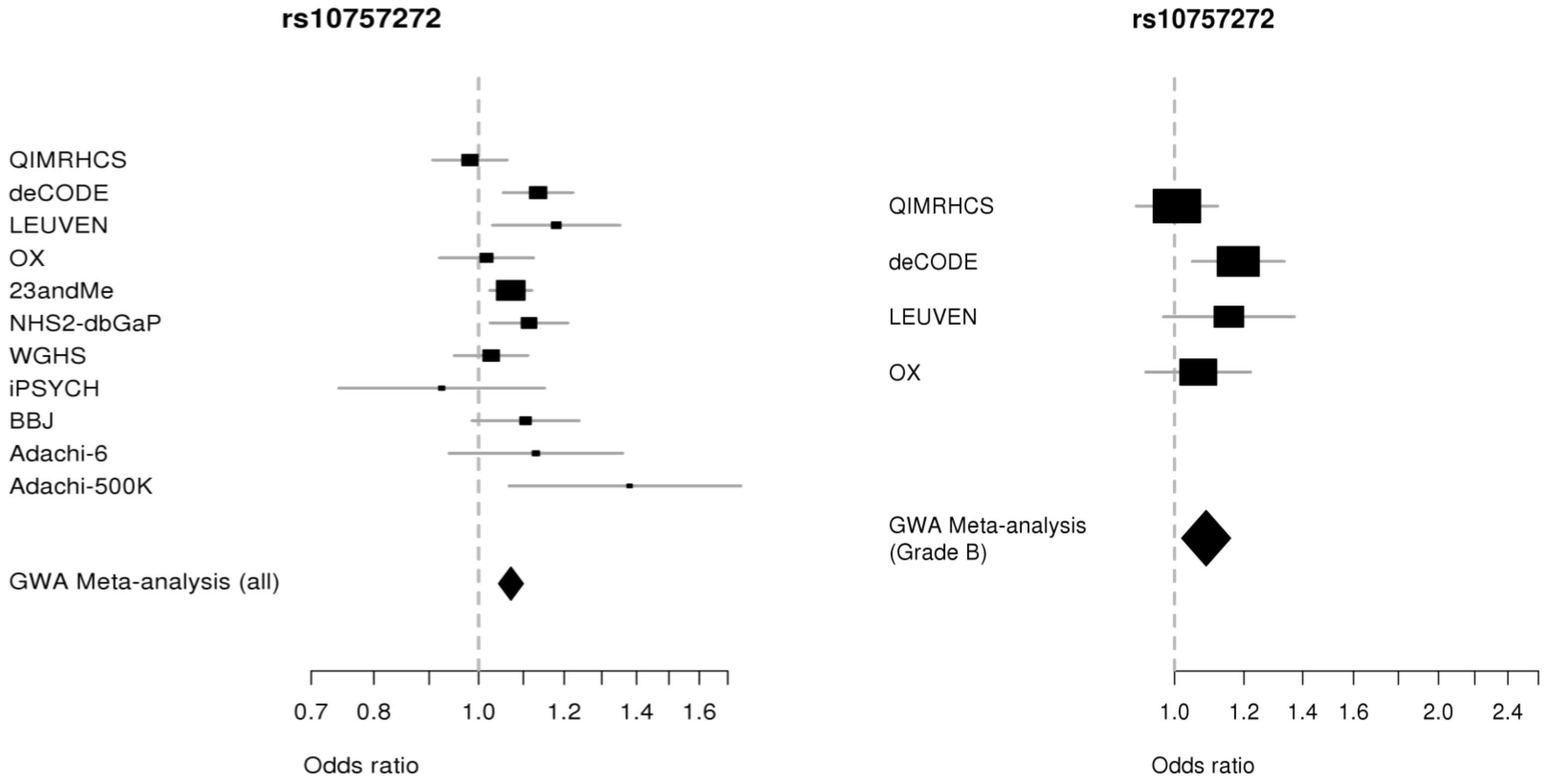

Supplementary Figure 41 | Forest plots of risk allele effects for a secondary association signal at 9p21.3 locus in the individual case-control datasets and GWA meta-analysis including all (left) and Grade B (right) endometriosis cases.

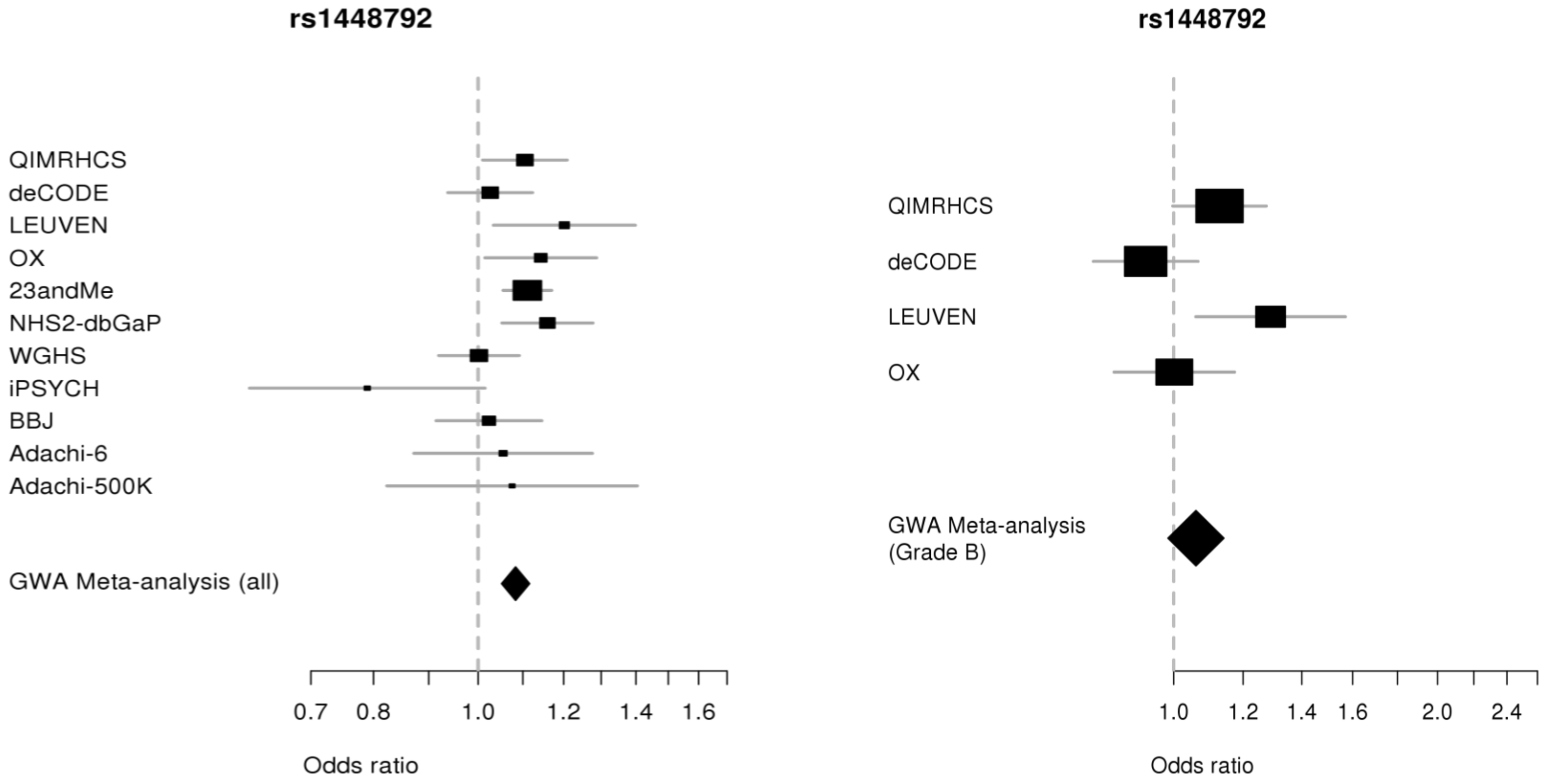

Supplementary Figure 42 | Forest plots of risk allele effects for a secondary association signal at 9p21.3 locus in the individual case-control datasets and GWA meta-analysis including all (left) and Grade B (right) endometriosis cases.

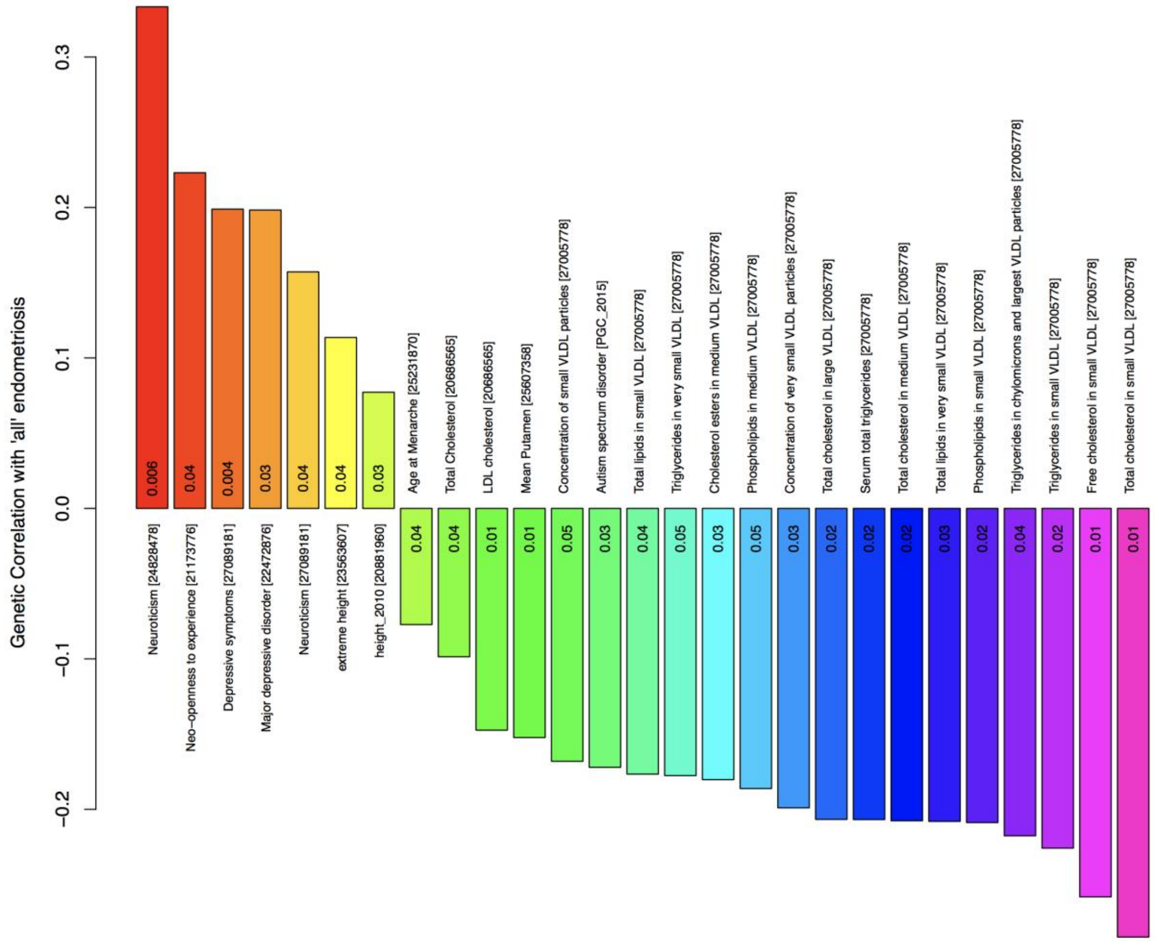

Supplementary Figure 43 | Barplots showing genetic correlation (y-axis) between all endometriosis and other traits with nominal $P < 0.05$, based on LD score regression analysis using LD hub. Numbers inside the Barplots are P values. PubMed ids of studies are shown inside parentheses.

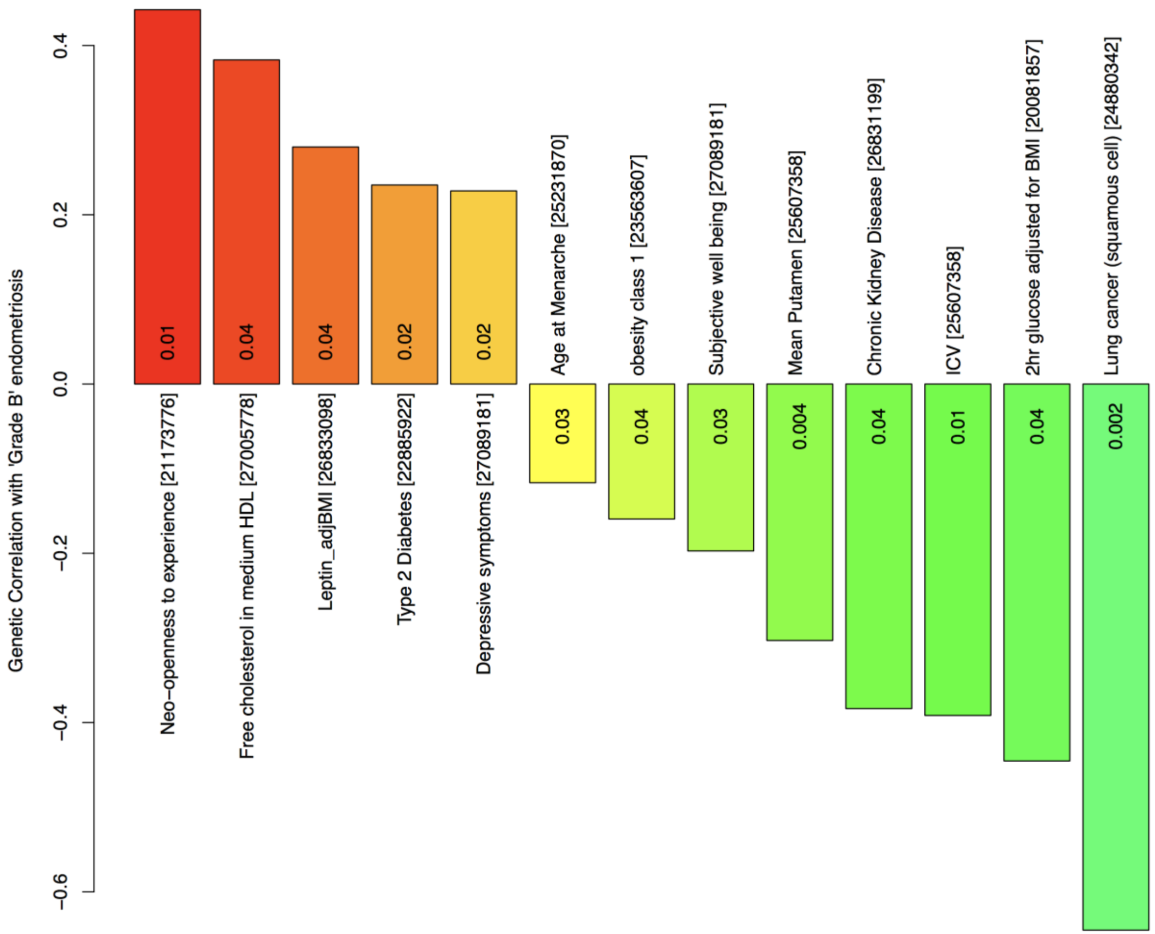

Supplementary Figure 44 | Barplots showing genetic correlation (y-axis) between Grade B endometriosis and other traits with nominal $P < 0.05$, based on LD score regression analysis using LD hub. Numbers inside the Barplots are P values. PubMed ids of studies are shown inside parentheses.

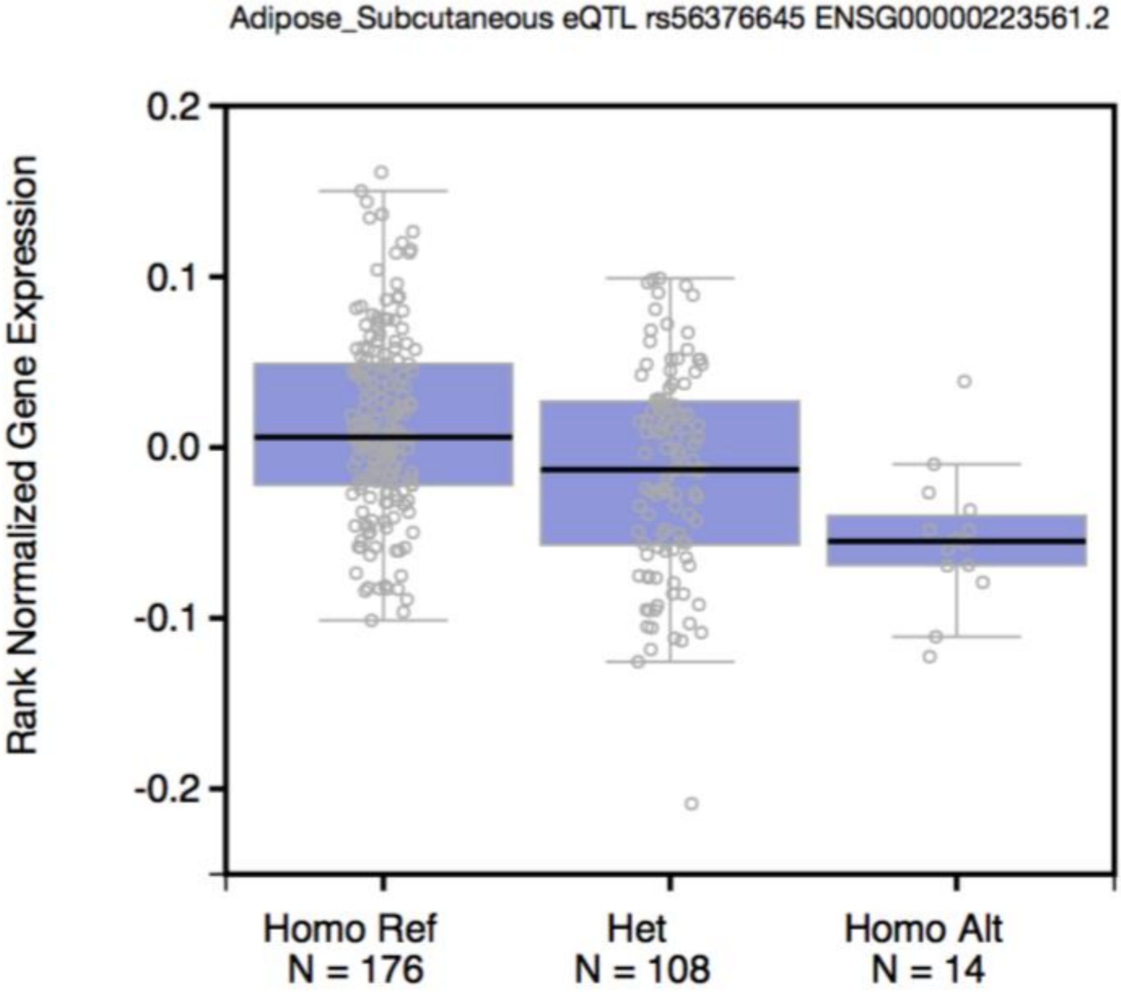

Supplementary Figure 45 | Box plot showing relationship between genotypes of rs56376645 and expression of *AC003090.1* in subcutaneous adipose tissue, based on GTEx Analysis Release V6 (dbGaP Accession phs000424.v6.p1).

Supplementary Note (GWA samples, genotyping and quality control)

QIMRHCS

Initially, 2,351 endometriosis patients were assessed from individuals recruited by The Queensland Institute of Medical Research (QIMR), Brisbane, Australia, between 1995-2002 (each completing a questionnaire and providing a blood sample). Surgical diagnosis for all endometriosis cases was confirmed from retrospective examination of medical records. Australian controls consisted of 1,870 individuals recruited by QIMR and 1,244 individuals recruited by the Hunter Community Study (HCS). Approval for the studies was obtained from the QIMR Human Ethics Research Committee, the University of Newcastle and Hunter New England Population Health Human Research Ethics Committees. Informed consent was obtained from all participants prior to testing.

QIMR cases and controls were genotyped at deCODE genetics on Illumina 670-Quad (cases) and 610-Quad (controls) Beadchips. HCS controls were genotyped at the University of Newcastle on 610-Quad Beadchips (Illumina). Genotypes for QIMR cases and controls were called with Illumina BeadStudio software. Standard quality control procedures were applied as outlined previously. Briefly, individuals with call rate of <0.95 and SNPs with mean BeadStudio GenCall score of <0.7, call rate of <0.95, Hardy-Weinberg equilibrium (HWE) P value of $<1 \times 10^{-6}$ or minor allele frequency (MAF) of <0.01 were excluded. Cryptic relatedness between individuals was identified through a full identity-by-state (IBS) matrix. Ancestry outliers were identified using data from 11 populations from HapMap 3 and 5 Northern European populations genotyped by the GenomeEUtwin Consortium using EIGENSOFT. To increase the power of the Australian GWAS data set, we matched the existing QIMR cases and controls by ancestry to individuals from the HCS genotyped on Illumina 610-Quad chips. After stringent quality control, the resulting QIMRHCS cohort consisted of 2,262 endometriosis cases and 2,924 controls.

OX

A total of 1,030 cases were drawn from women recruited by the Oxford Endometriosis Gene (OXEGENE) study. UK controls encompassed 6,000 individuals provided by the

WTCCC2. Informed consent was obtained from all participants and the study was approved by the Oxford regional multi-center and local research ethics committees.

OX cases were genotyped at deCODE genetics on Illumina 670-Quad and the WTCCC2 controls were genotyped at the Wellcome Trust Sanger Institute using Illumina HumanHap 1M Beadchips. Quality control procedures for the OX genotype data resulted in the removal of SNPs with genotype call rate of <0.99 and/or heterozygosity of <0.31 or >0.33. Genome-wide IBS was estimated for each pair of individuals, and one individual from each duplicate or related pair (IBS > 0.82) was removed. Genotype data were combined with data from the Utah residents of Northern and Western European ancestry (CEU), Han Chinese in Beijing, China (CHB) and Japanese in Tokyo, Japan (JPT), and Yoruba from Ibadan, Nigeria (YRI) HapMap 3 reference populations, and individuals who did not have Northern European ancestry were identified using EIGENSOFT and subsequently removed. SNPs with genotype call rate of <0.95 were removed, and this threshold was increased to 0.99 for SNPs with MAF of <0.05. In addition, SNPs showing (i) deviation from HWE ($P < 1 \times 10^{-6}$); (ii) difference in call rate between the 1958 British Birth Cohort (58BC) and National Blood Service (NBS) control groups ($P < 1 \times 10^{-4}$); (iii) difference in allele and/or genotype frequency between control groups ($P < 1 \times 10^{-4}$); (iv) difference in call rate between cases and controls ($P < 1 \times 10^{-4}$) and (v) MAF of <0.01 were removed.

The QIMR and Oxford studies used the same principles for confirmation of diagnosis and staging of disease, based on the revised American Fertility Society (rAFS) classification system. Disease severity was assessed retrospectively from medical records by use of the rAFS classification system, which assigns patients to one of four stages (I–IV) on the basis of the extent of disease and the associated adhesions present. As it can be difficult retrospectively to stage disease accurately using clinical records alone, a simplified two-stage system was used: Grade A (rAFS I/II disease or some ovarian disease plus a few adhesions) and Grade B (rAFS III/IV disease).

BBJ

All Japanese GWAS case and control samples were obtained from BioBank Japan at the Institute of Medical Science at the University of Tokyo. A total of 1,423 cases were

diagnosed with endometriosis by the presence of multiple clinical symptoms, physical examinations and/or laparoscopy or imaging tests. We used 1,318 female control samples from healthy volunteers from the Osaka-Midosuji Rotary Club (Osaka, Japan) and women in BioBank Japan who were registered to have no history of endometriosis. All participants provided written informed consent to this study. The study was approved by the ethical committees at the Institute of Medical Science at the University of Tokyo and the Center for Genomic Medicine at the RIKEN Yokohama Institute.

The BBJ cases and controls were genotyped using the Illumina HumanHap550v3 Genotyping BeadChip. Quality control filtering required sample call rate of ≥0.98, IBS analysis was used to exclude samples with close relatedness and principal-component analysis was used to exclude non-Asian samples. We also performed SNP quality control (call rate of ≥0.99 in both cases and controls and HWE P of $\geq 1 \times 10^{-6}$ in controls). In total, 460,945 SNPs on all chromosomes passed the quality control filters and were further analysed.

NHS2-dbGaP

NHS2 endometriosis cases were drawn from US Nurses' Health Study (NHS) II, a prospective cohort study with follow-up from 1989-2007. Biennially, 116,678 registered female nurses – aged 25-53 and residing in 14 of the US states – complete questionnaire information on incidence of disease outcomes and biological, environmental, dietary, and life-style risk factors. From 1996-1998, blood samples were collected from 29,613 participants 32-53 years of age. Women were asked if they had "ever had physician-diagnosed endometriosis", the date of diagnosis, and whether diagnosis had been confirmed by laparoscopy. To assess the validity of self-reported endometriosis, the laparoscopy records of 200 randomly selected women who had reported a diagnosis from 1989-1993 were sought; the diagnosis was confirmed in 96% of 105 women who had surgery and whose records were available. The NHS2 case dataset comprised 2,400 cases with a self-reported laparoscopy-confirmed diagnosis of endometriosis and available blood samples, all of self-reported European descent. Participant enrolment, questionnaire and clinical data, and biologic sample collection have been approved by the Human Subject Committee of Harvard School of Public Health and by the Institutional Review Board of Brigham and Women's Hospital.

Controls were from the Genetic Epidemiology of Chronic obstructive pulmonary disease (COPDGene) study obtained through dbGaP (Study Accession: phs000179.v4.p2). A total of 10,000 subjects will be recruited, including control smokers, definite COPD cases (GOLD Stage 2 to 4), and subjects not included in either group (GOLD 1 or GOLD-Unclassified). The primary focus of the COPDGene study was genome-wide association analysis to identify the genetic risk factors that determine susceptibility for COPD and COPD-related phenotypes. The dbGaP controls used in this study included 2,490 non-hispanic white control smokers, with age 45-80 years, smoking history (current or formerly) of ≥ 10 pack-years, absence of COPD and uncontrolled cancer, defined as ongoing radiation therapy, ongoing chemotherapy, narcotics for pain control, or known metastatic disease, etc. (see http://www.ncbi.nlm.nih.gov/projects/gap/cgi-bin/study.cgi?study_id=phs000179.v4.p2 for more details).

NHS2 cases were genotyped at QIMR Berghofer Medical Research Institute, Brisbane on Illumina HumanCoreExome-12v1.0 array, with ~250,000 common tag SNPs, whereas dbGaP controls were genotyped on HumanOmni1-Quadv1.0. Quality control for the NHS2-dbGaP GWA data resulted in the removal of SNPs with >1% missing rate, HWE $P < 1 \times 10^{-6}$ in controls and MAF <0.01. Similarly, individuals with >1% missing rate, outlying heterozygosity (+/- three standard deviations from the mean) and of non-European ancestry based on the 1000G reference population were excluded as was one individual from each duplicate or related (pi-hat >0.2). After stringent quality controls, the NHS2-dbGaP dataset resulted in 2,238 endometriosis cases and 2,317 controls.

Adachi

All women with endometriosis were registered at the Niigata University Hospital, the Nagasaki University Hospital, the Kumamoto University Hospital, the Takarazuka City Hospital, the National Hospital Organization Kyoto Medical Center and several hospitals in Niigata, Toyama and Yamagata Prefecture (for details, see Acknowledgements). The affected subjects comprised 728 Japanese women who fulfilled any of the following diagnostic criteria: (i) women who underwent laparotomy or laparoscopic surgery for diseases other than endometriosis, each of which procedure was also used to provide biopsy proven evidence of endometriosis, (ii) women who were verified to have endometriosis in diagnostic laparoscopy and (iii) women who were diagnosed to have

ovarian cysts by imaging diagnostics. Control samples for association analysis comprised 834 Japanese women from various resources as follows: (i) 96 fertile women or those with benign gynecological tumors, with no history of endometriosis diagnosed at the Niigata University Hospital, (ii) 241 females from the Japanese Integrated Database Project (81 from panic disorder control cohort, 77 from multiple system atrophy control cohort and 83 from control database cohort), and (iii) 497 females from late-onset Alzheimer's disease control cohort. Informed consent was obtained from all the participants and the study was approved by the ethical committee of the University of Niigata and the affiliated hospitals.

Genotyping was conducted at Tokai University and Niigata University on two types of Affymetrix platforms, Gene-Chip Human Mapping 500K array or Genome-Wide Human SNP array 6.0 (Affymetrix, Santa Clara, CA, USA), according to the manufacturer's instruction. The 411 subjects (315 cases and 96 controls with fertility or no endometriosis) were newly genotyped with 500K arrays, and 413 subjects (413 cases) were genotyped with 6.0 arrays. Genotype calls were determined with the Bayesian Robust Linear Model using Mahalanobis distance classifier (BRLMM) algorithm21 for 500K arrays or the Birdseed v2 algorithm for 6.0 arrays, embedded in Affymetrix Genotyping Console 3.0.1. Quality control excluded samples in each of array cohorts that showed >5% missing genotypes, outliers with respect to genome-wide heterozygosity (<21% or >30% heterozygous SNP rate), and population outliers based on 90 HapMap samples (45 JPT and 45 CHB samples). One individual from the duplicate or related pair showing cryptic relatedness based on pairwise IBS analysis was also excluded. After the above sample quality control, the remaining 497 control samples from late-onset Alzheimer's disease control cohort, which had been independently quality controlled, were added to perform the following SNP quality control in each of array cohorts. Markers were excluded with MAF <0.01 in cases and controls, ≥4% (for 500K array) and ≥2% (for 6.0 array) missing rate in cases and controls, exact test *P* value <0.05 for the missing rate differences between cases and controls, and HWE exact *P* value $<10^{-5}$ in cases and controls. After stringent quality controls, the Adachi dataset included 696 endometriosis cases and 825 controls.

23andMe

The 23andMe endometriosis cohort consisted of 4,970 endometriosis cases and 34,561 controls assessed from the 23andMe GWA participant cohort. Diagnosis of endometriosis was based on the self-reported questionnaires related to participants' including a survey on medical history, a survey on female fertility, and single questions on endometriosis. All research participants provided informed consent and answered surveys online according to 23andMe's human subjects protocol, which was reviewed and approved by Ethical & Independent Review Services, a private institutional review board (http://www.eandireview.com).

DNA extraction and genotyping were performed on saliva samples by National Genetics Institute (NGI), a CLIA-certified laboratory and a subsidiary of Laboratory Corporation of America. Samples have been genotyped on one of three genotyping platforms. The V1 and V2 platforms were variants of the Illumina HumanHap550+ BeadChip, including about 25,000 custom SNPs selected by 23andMe, with a total of about 560,000 SNPs. The V3 platform was based on the Illumina OmniExpress+ BeadChip, with custom content to improve the overlap with our V2 array, with a total of about 950,000 SNPs. All research participants were women who had >97% European ancestry, as determined through an analysis of local ancestry via comparison to the three HapMap 2 populations. A maximal set of unrelated individuals was chosen for the analysis using a segmental identity-by-descent (IBD) estimation algorithm. Individuals were defined as related if they shared more than 700 cM IBD, including regions where the two individuals share either one or both genomic segments identical-by-descent. This level of relatedness (roughly 20% of the genome) corresponds approximately to the minimal expected sharing between first cousins in an outbred population. Samples that failed to reach 98.5% call rate were reanalyzed. Participants whose sample analyses failed repeatedly were re-contacted by 23andMe customer service to provide additional samples. SNPs with MAF <0.001, HWE $P < 10^{-20}$, call rate < 95%, or with large allele frequency discrepancies compared to the 1000 Genomes reference data were excluded.

WGHS

The Women's Genome Health Study (WGHS) is a prospective cohort of initially healthy, female North American health care professionals at least 45 years old at baseline representing participants in the Women's Health Study (WHS) who provided a blood

sample at baseline and consent for blood-based analyses. The WHS was a 2x2 trial beginning in 1992-1994 of vitamin E and low dose aspirin in prevention of cancer and cardiovascular disease with about 10 years of follow-up. Since the end of the trial, follow-up has continued in observational mode. Additional information related to health and lifestyle were collected by questionnaire throughout the WHS trial and continuing observational follow-up.

Endometriosis status was ascertained by the eighth questionnaire during the observational follow-up. WHS participants were asked: ”Have you EVER had physician-diagnosed endometriosis (yes/no)? If yes, has your endometriosis diagnosis been confirmed by laparoscopy (a standard method for diagnosing endometriosis) (yes/no/unsure)?" From these questions, endometriosis status was defined two ways. A stringent definition specified cases who responded “yes” to both questions. All other endometriosis cases were defined by response “yes” to the first question, and a response of “no”, “unsure”, or “missing” to the second question. Controls were defined as responding “no” to the first question. These controls were equally divided at random into two groups for pairing with the two sets of cases such that no participants were shared by analyses of two case definitions.

iPSYCH

A total of 205 cases and 930 age-matched controls were drawn from the iPSYCH sample, which is a population-based sample of more than 80,000 individuals derived from the Danish Neonatal Screening Biobank. In short the Danish Neonatal Screening Biobank comprise dried bloodspots (Guthrie cards) from all individuals born in Denmark since 1981, where DNA extracted from the bloodspots can be successfully amplified and employed in GWAS[1]. All samples can be linked to the Danish register system, including the Danish National Patient Register (DNPR) that contains information about all hospital admissions and discharge, diagnoses and surgical procedures since 1977[2]. The approximately 80,000 iPSYCH samples were drawn to study mental disorders, including approximately 30,000 population-based controls. The endometriosis cases were identified from the entire iPSYCH sample using ICD-8: 625.30–39 and ICD-10: N80.0–N80.9 (the 9th revision of ICD was not used in Denmark) from the DNPR. The control individuals were selected to be age-matched individuals from the iPSYCH controls. All

endometriosis cases in the iPSYCH sample were younger than 32 years. Genotyping was performed at the Broad Institute using the Infinium PsychArray. Samples were genotyped in 24 waves. Quality control and SNP filtering was performed using the Ricopili pipeline described previously[3]. In short, genotype calling was performed using a combination of Autocall, Birdseed and Zcall algorithm. SNPs with call rate < 98%, MAF <0.01, deviation from HWE ($P < 1 \times 10^{-6}$) among controls were removed. After stringent quality controls, the iPSYCH dataset included 205 endometriosis cases and 930 controls of European descent. This study has been approved by the Danish research ethical committee system.

deCODE

Endometriosis cases and controls from deCODE were from the deCODE Genetics, Iceland. Genome-wide imputation of this cohort was based on whole genome sequencing of 8,453 Icelanders using Illumina technology to a mean depth of at least 10X (median 32X). Approximately 30 million sequenced variants were then imputed into 150,656 Icelanders who had been genotyped using Illumina genotyping arrays. Using genealogic information, the sequence variants were imputed into 294,212 un-typed relatives of chip-typed individuals to further increase the sample size for association analysis and increased the power to detect associations. Of these, this cohort included 1,840 surgically diagnosed endometriosis cases and 129,016 controls, with 688 cases exhibiting moderate-to-severe (rAFS III or IV) disease.

Reference

1. Hollegaard, M.V. *et al.* Robustness of genome-wide scanning using archived dried blood spot samples as a DNA source. *BMC Genet* **12**, 58 (2011).

2. Lynge, E., Sandegaard, J.L. & Rebolj, M. The Danish National Patient Register. *Scand J Public Health* **39**, 30-3 (2011).

3. Schizophrenia Working Group of the Psychiatric Genomics, C. Biological insights from 108 schizophrenia-associated genetic loci. *Nature* **511**, 421-7 (2014).
